# Thermal Contact Response of a Transversely Isotropic Magneto-Electro-Elastic Coating

**DOI:** 10.3390/ma17010128

**Published:** 2023-12-26

**Authors:** Yutang Li, Cenbo Xiong, Qinghua Zhou, Wanyou Yang, Rongsong Yang

**Affiliations:** 1 School of Mechanical Engineering, Beijing Institute of Technology, Beijing 100081, China; liyutanglyt@163.com; 2 School of Mechanical Engineering, Sichuan University, Chengdu 610065, China; 13981903849@139.com; 3 Sichuan Aerospace Changzheng Equipment Manufacturing Co., Ltd., Chengdu 610100, China; 4 School of Aeronautics and Astronautics, Sichuan University, Chengdu 610065, China; qh.zhou@foxmail.com; 5 School of Aeronautics and Astronautics, University of Electronic Science and Technology of China, Chengdu 611731, China; wanyou.yang@foxmail.com

**Keywords:** thermal contact, magneto-electro-elasticity, coating, semi-analytical method

## Abstract

The magneto-electro-elastic (MEE) medium is a typical intelligent material with promising application prospects in sensors and transducers, whose thermal contact response is responsible for their sensitivity and stability. An effective thermal contact model between a moving sphere and a coated MEE medium with transverse isotropy is established via a semi-analytical method (SAM) to explore its thermal contact response. First, a group of frequency response functions for the magneto-electro-thermo-elastic field of a coated medium are derived, assuming that the coating is perfectly bonded to the substrate. Then, with the aid of the discrete convolution–fast Fourier transform algorithm and conjugate gradient method, the contact pressure and heat flux can be determined. Subsequently, the induced elastic, thermal, electric and magnetic fields in the coating and substrate can be obtained via influence coefficients relating the induced field and external loads. With the proposed method, parametric studies on the influence of the sliding velocity and coating property are conducted to investigate the thermal contact behavior and resulting field responses of the MEE material. The sliding velocity and thermal properties of the coating have a significant effect on the thermal contact response of the MEE material; the coupled multi-field response can be controlled by changing the coating thickness between ~0.1 a_0_ and a_0_.

## 1. Introduction

Owing to the multiple excellent coupled properties of mechanics, electricity and magnetism, a magneto-electro-elastic (MEE) material can convert energy from one to the other, making it widely used in sensors, transducers, generators, and medical equipment as an intelligent structure [1,2,3,4]. In practical engineering applications, the MEE material is usually applied in sensing and driving devices in the form of a thin film or layered structure [5,6,7], where friction contact occurs inevitably on its surface [8]. Friction contact is frequently accompanied by friction heat, making it necessary to take the physical discontinuity of the layered structure and the material’s transverse isotropy [9] into consideration, which affects its mechanical and electromagnetic coupling properties significantly [10,11]. Therefore, an effective thermal contact model of a coated MEE medium is valuable in analyzing the multi-physical field response, providing theoretical guidance for engineering applications of the MEE layered structure.

Research on layered MEE materials has been ongoing for a long time, and many attempts at theoretical general solution derivation, contact modeling, and damage analysis have been made. A general solution is an effective method of evaluating the field response of an MEE material, with high calculation efficiency and solid mathematical bases. For instance, Mousavi and Paavola analytically obtained closed-form expressions of the shear stress, electric displacement and magnetic induction in a functionally graded, coated MEE medium by using Fourier transform technology, which was applied to solve the damage problem of the coated MEE medium [12]. Li and Pan derived the analytical solution for an anisotropic multilayer MEE medium and studied the multi-field coupling response caused by the traction force and dislocation in a multilayer structure [13]. In addition to the analytical method used in the work mentioned above [12,13], the asymptotic homogenization method is another effective method of deriving a general solution for MEE materials [14]. With the asymptotic homogenization method, Sixto-Camacho et al. [15] developed the formal asymptotic solution for the linear magneto-electro-thermo-elastic field of heterogeneous media. Combining the asymptotic homogenization method and the cell-based smoothed finite element method, Zhou et al. [16] established a multi-physics coupling model for an MEE structure and the transient responses under dynamic loads were investigated. Different from those focusing on the multi-physical field, Chaki and Bravo-Castillero [17] studied wave propagation in an MEE laminated structure via dynamic asymptotic homogenization. Once the tribological behavior between bodies is considered, contact modeling is necessary to analyze the effects of friction. Some studies have focused on the contact responses due to the mechanical load and material parameters. For example, Zhang et al. established a semi-analytical model of the dynamic contact between a rigid ball and MEE film, and they analyzed the effect of the loading speed, film thickness and ball radius on the dynamic magneto-electro-elastic response [18]. Zhang et al. proposed a contact model of a functionally graded, coated MEE medium and studied the effects of the coating thickness and coating parameters on the elastic, electric and magnetic fields of the coated medium [19]. Some have also paid attention to the effects of the electromagnetic field on the contact response. Sui et al. [20] established a semi-analytical contact model for a 3D MEE material and found that the electric field can control the magnetic field via strain transfer but not vice versa. Under external loading, stress concentration means that damage to the material unavoidably occurs, becoming a concern for some researchers. Wan et al. studied the periodic interface damage problem of multilayer piezoelectric/piezomagnetic composites subjected to electric and magnetic loads, and they analyzed the effect of the material parameters on the stress intensity factor [21]. Arhani and Ayatollahi [22] derived an analytical solution for MEE dislocation in a cracked, functionally graded MEE material and investigated the dynamic stress intensity factor.

Sliding contact is usually accompanied by frictional heating on the contact interface; however, the thermal effect on the MEE material was not taken into account in the previous work mentioned above. It has been found that the thermal effect also affects the multi-field coupling effect of the MEE material [23,24,25], attracting increasing attention. Chen et al. derived the general solution for the elastic, electric, magnetic and temperature fields of an MEE material by considering the thermal effect, and the solution was used to solve the crack problem in infinite space [26]. Zhou et al. carried out research on the multi-field coupled response of an MEE cylindrical shell and plate structure undergoing a thermal effect and revealed the static characteristic of the MEE plate structure under a thermal load using the finite element method [27]. Similarly, Ni et al. deduced the analytical solution for an MEE cylindrical shell with a thermal effect and studied the influence of the geometric parameters, material volume fraction and external electric/magnetic/thermal loads on the buckling stresses and mode shapes of the shell structure [28]. Further considering the existence of a crack in the cylinder, Chang et al. [29] obtained exact solutions for the prediction of magneto-electro-thermo-elastic fields under thermal shock. The asymptotic homogenization method is also a good candidate for a solution for MEE materials when considering the thermal effect. Bravo-Castillero et al. [30] used the asymptotic homogenization method to study the three-dimensional boundary values of MEE composites when considering the thermal effect. Similar to the asymptotic homogenization method, a symbolic mathematics approach was used to derive quasi-harmonic solutions for MEE materials with transverse isotropy by Marmo and Francesco [31]. Regarding thermal contact for the MEE material, Çömez [32] developed a thermal contact model for a two-dimensional MEE layer, where the punches were treated as thermal insulators.

The above research on MEE materials considering the thermal effect has mainly focused on the mechanical and the electric and magnetic responses of the cylindrical and disk structures. Few studies have focused on the thermal contact behavior of the MEE coating. Although a thermal contact model for the two-dimensional MEE layer has been reported, this is still a problem for the half plane, where the heat partition at the contact interface has not been considered. Therefore, this paper puts forward an effective three-dimensional thermal contact model of the coated MEE medium considering heat partition, aiming to reveal the effects of the sliding velocity, thermal parameters and coating thickness on the coupled multiple physical fields (mechanics, electricity, magnetism and temperature). The main content of this work includes (a) the derivation of the frequency response functions (FRFs) of the coupled physical fields of the coated MEE medium considering the thermal effects; (b) the establishment of the thermal contact model between a sliding ball and the coated MEE medium considering heat partition; (c) the verification of the proposed model by comparing the results obtained from the finite element method; (d) the investigation of the effects of the sliding velocity and coating parameters on the mechanical, electromagnetic and temperature rise responses of the coated MEE medium.

## 2. Basic Formulation

### 2.1. Problem Description

**Figure 1:**
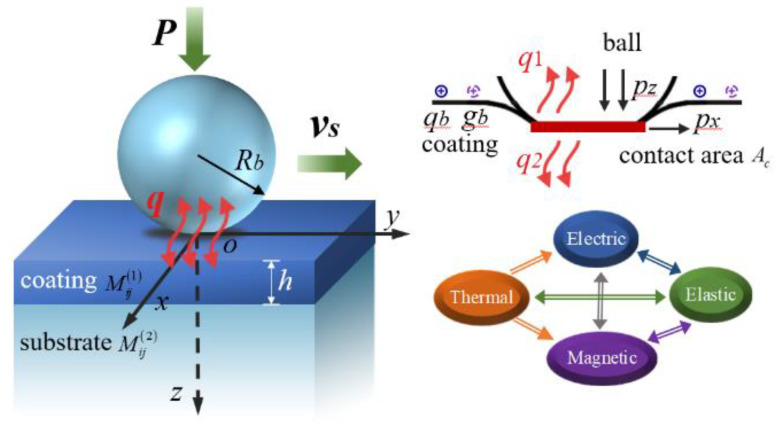
Thermal contact model between a loaded sliding ball and the coated MEE medium.

Figure 1 presents a thermal contact model between a loaded sliding ball and a transversely isotropic coated infinite half-space composed of the magneto-electro-elastic (MEE) material. A Cartesian coordinate system is established, where the coating surface is set as the *x*–*y* plane. *R_b_* represents the radius of the sliding ball, and *h* represents the thickness of the thin solid film that is perfectly bonded on the substrate. Both of the two contact bodies are composed of the MEE material, and the material properties of the ball are set to be the same as those of the substrate, while the material parameters of the coating are alterable. The contact ball is subjected to a normal load *P* sliding on the coating surface with a velocity *v*_s_ along the *x* axis. Due to the friction contact effects, the contact pressure *p_z_*, the traction *p_x_* and the heat flux *q* are thus generated on the coating surface. It is assumed that all of the frictional work is converted to heat completely, and then flows into the ball (*q*_1_) and the coated MEE medium (*q*_2_) through the contact area without heat dissipation. *q_b_*, *g_b_* are the electric and the magnetic charge distributed on the coating surface, respectively. Here, the transversely isotropic coated MEE medium is subjected to multiple surface loads (*p_z_*, *p_x_*, *q*_2_, *q_b_* and *g_b_*), resulting in elastic, thermal, electric and magnetic coupled multi-field responses in both the coating and substrate.

### 2.2. Basic Formulation

Both the coating and the substrate are composed of the transversely isotropic MEE material, and their constitutive relations are given as follows [18]:

(1) $$\begin{array}{l} \sigma_{xx}=c_{11}\frac{\partial u_{x}}{\partial x}+c_{12}\frac{\partial u_{y}}{\partial y}+c_{13}\frac{\partial u_{z}}{\partial z}+e_{31}\frac{\partial \phi }{\partial z}+d_{31}\frac{\partial \psi }{\partial z}-\beta_{1}T,\\ \sigma_{yy}=c_{12}\frac{\partial u_{x}}{\partial x}+c_{11}\frac{\partial u_{y}}{\partial y}+c_{13}\frac{\partial u_{z}}{\partial z}+e_{31}\frac{\partial \phi }{\partial z}+d_{31}\frac{\partial \psi }{\partial z}-\beta_{1}T,\\ \sigma_{zz}=c_{13}\frac{\partial u_{x}}{\partial x}+c_{13}\frac{\partial u_{y}}{\partial y}+c_{33}\frac{\partial u_{z}}{\partial z}+e_{33}\frac{\partial \phi }{\partial z}+d_{33}\frac{\partial \psi }{\partial z}-\beta_{3}T,\\ \sigma_{xz}=c_{44}\left(\frac{\partial u_{x}}{\partial z}+\frac{\partial u_{z}}{\partial x}\right)+e_{15}\frac{\partial \phi }{\partial x}+d_{15}\frac{\partial \psi }{\partial x},\\ \sigma_{yz}=c_{44}\left(\frac{\partial u_{y}}{\partial z}+\frac{\partial u_{z}}{\partial y}\right)+e_{15}\frac{\partial \phi }{\partial y}+d_{15}\frac{\partial \psi }{\partial y},\sigma_{xy}=c_{66}\left(\frac{\partial u_{x}}{\partial y}+\frac{\partial u_{y}}{\partial x}\right), \end{array}$$

(2) $$\begin{array}{l} D_{x}=e_{15}\left(\frac{\partial u_{x}}{\partial z}+\frac{\partial u_{z}}{\partial x}\right)-\varepsilon_{11}\frac{\partial \phi }{\partial x}-g_{11}\frac{\partial \psi }{\partial x},\\ D_{y}=e_{15}\left(\frac{\partial u_{y}}{\partial z}+\frac{\partial u_{z}}{\partial y}\right)-\varepsilon_{11}\frac{\partial \phi }{\partial y}-g_{11}\frac{\partial \psi }{\partial y},\\ D_{z}=e_{31}\frac{\partial u_{x}}{\partial x}+e_{31}\frac{\partial u_{y}}{\partial y}+e_{33}\frac{\partial u_{z}}{\partial z}-\varepsilon_{33}\frac{\partial \phi }{\partial z}-g_{33}\frac{\partial \psi }{\partial y}+p_{3}T, \end{array}$$

(3) $$\begin{array}{l} B_{x}=d_{15}\left(\frac{\partial u_{x}}{\partial z}+\frac{\partial u_{z}}{\partial x}\right)-g_{11}\frac{\partial \phi }{\partial x}-\mu_{11}\frac{\partial \psi }{\partial x},\\ B_{y}=d_{15}\left(\frac{\partial u_{y}}{\partial z}+\frac{\partial u_{z}}{\partial y}\right)-g_{11}\frac{\partial \phi }{\partial y}-\mu_{11}\frac{\partial \psi }{\partial y},\\ B_{z}=d_{31}\frac{\partial u_{x}}{\partial x}+d_{31}\frac{\partial u_{y}}{\partial y}+d_{33}\frac{\partial u_{z}}{\partial z}-g_{33}\frac{\partial \phi }{\partial z}-\mu_{33}\frac{\partial \psi }{\partial y}+\lambda_{3}T, \end{array}$$

where ***σ***, ***D*** and ***B*** denote the mechanical stresses, the electric displacement and the magnetic induction, respectively; ***u***, ***ϕ***, ***ψ*** and ***T*** are the displacement, electric potential, magnetic potential and temperature rise; *c_ij_*, *ε_ij_*, *e_ij_*, *q_ij_*, *d_ij_*, *μ_ij_*, *p*_3_ and *λ*_3_ represent the elastic, dielectric, piezoelectric, piezomagnetic, magnetoelectric, magnetic, pyroelectric and pyromagnetic constants of the material, respectively. Note that *β_i_* stands for the thermal modulus related to the thermal expansion *α_i_* in Ref. [25], and $c_{11}=c_{12}+2c_{66}$.

In the absence of body sources, the equilibrium equations, the Maxwell equations and the heat conduction equations [33] are

(4) $$\begin{array}{l} \frac{\partial \sigma_{x}}{\partial x}+\frac{\partial \tau_{xy}}{\partial y}+\frac{\partial \tau_{xz}}{\partial z}=0,\\ \frac{\partial \tau_{xy}}{\partial x}+\frac{\partial \sigma_{y}}{\partial y}+\frac{\partial \tau_{yz}}{\partial z}=0,\\ \frac{\partial \tau_{xz}}{\partial x}+\frac{\partial \tau_{yz}}{\partial y}+\frac{\partial \sigma_{z}}{\partial z}=0, \end{array}$$

(5) $$\begin{array}{l} \frac{\partial D_{x}}{\partial x}+\frac{\partial D_{y}}{\partial y}+\frac{\partial D_{z}}{\partial z}=0,\\ \frac{\partial B_{x}}{\partial x}+\frac{\partial B_{y}}{\partial y}+\frac{\partial B_{z}}{\partial z}=0, \end{array}$$

(6) $$\left(\frac{\partial^{2}}{\partial x^{2}}+\frac{\partial^{2}}{\partial y^{2}}+\frac{k_{33}}{k_{11}}\frac{\partial^{2}}{\partial z^{2}}+Pe\frac{\partial }{\partial x}\right)T=0,$$

where *k_ij_* are the heat conductivities, *Pe* = *v_s_l_s_*/(*k*_11_/*c*_h_) is the Peclet number, *l_s_* is the characteristic contact length, and *c*_h_ represents the volumetric specific heat.

Substituting Equations (1)–(3) into Equations (4)–(6), the equilibrium equations can be expressed in terms of *Ψ*, *G*, *u_z_*, ***ϕ***, ***ψ***, ***T***:

(7) $$\left(c_{66}\Delta +c_{44}\frac{\partial^{2}}{\partial z^{2}}\right)\Psi =0,$$

(8) $$D\left[\begin{array}{c} G\\ u_{z}\\ \phi \\ \psi \\ T \end{array}\right]=\left[\begin{array}{c} 0\\ 0\\ 0\\ 0\\ 0 \end{array}\right],$$

(9) $$D=\left[\begin{array}{ccccc} c_{11}\Delta +c_{44}\frac{\partial^{2}}{\partial z^{2}} & -\left(c_{13}+c_{44}\right)\frac{\partial }{\partial z} & -\left(e_{15}+e_{31}\right)\frac{\partial }{\partial z} & -\left(d_{15}+d_{31}\right)\frac{\partial }{\partial z} & \beta_{1}\\ -\left(c_{13}+c_{44}\right)\Delta \frac{\partial }{\partial z} & c_{44}\Delta +c_{33}\frac{\partial^{2}}{\partial z^{2}} & e_{15}\Delta +e_{33}\frac{\partial^{2}}{\partial z^{2}} & d_{15}\Delta +d_{33}\frac{\partial^{2}}{\partial z^{2}} & -\beta_{3}\frac{\partial }{\partial z}\\ \left(e_{15}+e_{31}\right)\Delta \frac{\partial }{\partial z} & -\left(e_{15}\Delta +e_{33}\frac{\partial^{2}}{\partial z^{2}}\right) & \varepsilon_{11}\Delta +\varepsilon_{33}\frac{\partial^{2}}{\partial z^{2}} & g_{11}\Delta +g_{33}\frac{\partial^{2}}{\partial z^{2}} & -p_{3}\frac{\partial }{\partial z}\\ \left(d_{15}+d_{31}\right)\Delta \frac{\partial }{\partial z} & -\left(d_{15}\Delta +d_{33}\frac{\partial^{2}}{\partial z^{2}}\right) & g_{11}\Delta +g_{33}\frac{\partial^{2}}{\partial z^{2}} & \mu_{11}\Delta +\mu_{33}\frac{\partial^{2}}{\partial z^{2}} & -\lambda_{3}\frac{\partial }{\partial z}\\ 0 & 0 & 0 & 0 & \Delta +\frac{k_{33}}{k_{11}}\frac{\partial^{2}}{\partial z^{2}}+Pe\frac{\partial }{\partial x} \end{array}\right]$$

in which Δ = ∂^2^/∂*x*^2^ + ∂^2^/∂*y*^2^ is the two-dimensional Laplacian operator, ***D*** represents a differential operator matrix, and *Ψ* and *G* are two intermediate functions to simplify the expressions of Equations (1)–(3), defined as

(10) $$u_{x}=\frac{\partial \Psi }{\partial y}-\frac{\partial G}{\partial x},u_{y}=-\frac{\partial \Psi }{\partial x}-\frac{\partial G}{\partial y}.$$

Following the work of Chen et al. [18], the general solutions of the displacement, electric potential, magnetic potential and temperature rise can be obtained by operator theory as follows:

(11) $$\begin{array}{l} u_{x}=\frac{\partial \psi }{\partial y}-\left[a_{1}\frac{\partial^{6}}{\partial z^{6}}+b_{1}\Delta \frac{\partial^{4}}{\partial z^{4}}+f_{1}\Delta^{2}\frac{\partial^{2}}{\partial z^{2}}+g_{1}\Delta^{3}\right]\frac{\partial F}{\partial x},\;\\ u_{y}=\frac{\partial \psi }{\partial x}-\left[a_{1}\frac{\partial^{6}}{\partial z^{6}}+b_{1}\Delta \frac{\partial^{4}}{\partial z^{4}}+f_{1}\Delta^{2}\frac{\partial^{2}}{\partial z^{2}}+g_{1}\Delta^{3}\right]\frac{\partial F}{\partial y},\\ u_{z}=\left[a_{2}\frac{\partial^{6}}{\partial z^{6}}+b_{2}\Delta \frac{\partial^{4}}{\partial z^{4}}+f_{2}\Delta^{2}\frac{\partial^{2}}{\partial z^{2}}+g_{2}\Delta^{3}\right]\frac{\partial F}{\partial z},\\ \phi =\left[a_{3}\frac{\partial^{6}}{\partial z^{6}}+b_{3}\Delta \frac{\partial^{4}}{\partial z^{4}}+f_{3}\Delta^{2}\frac{\partial^{2}}{\partial z^{2}}+g_{3}\Delta^{3}\right]\frac{\partial F}{\partial z},\\ \varphi =\left[a_{4}\frac{\partial^{6}}{\partial z^{6}}+b_{4}\Delta \frac{\partial^{4}}{\partial z^{4}}+f_{4}\Delta^{2}\frac{\partial^{2}}{\partial z^{2}}+g_{4}\Delta^{3}\right]\frac{\partial F}{\partial z},\\ T=\left[n_{0}\frac{\partial^{8}}{\partial z^{8}}+n_{1}\Delta \frac{\partial^{6}}{\partial z^{6}}+n_{2}\Delta^{2}\frac{\partial^{4}}{\partial z^{4}}+n_{3}\Delta^{3}\frac{\partial^{2}}{\partial z^{2}}+n_{4}\Delta^{4}\right]F, \end{array}$$

where the coefficients *a_i_*, *b_i_*, *f_i_*, *g_i_* and *n_i_* can be found in Ref. [18], and the functions *Ψ* and *F* need to satisfy the following equations:

(12) $$\left(\Delta +\frac{\partial^{2}}{\partial s_{0}^{2}z^{2}}\right)\Psi =0,\;$$

(13) $$\left(\Delta +\frac{\partial^{2}}{\partial s_{1}^{2}z^{2}}\right)\left(\Delta +\frac{\partial^{2}}{\partial s_{2}^{2}z^{2}}\right)\left(\Delta +\frac{\partial^{2}}{\partial s_{3}^{2}z^{2}}\right)\left(\Delta +\frac{\partial^{2}}{\partial s_{4}^{2}z^{2}}\right)\left(\Delta +\frac{\partial }{\partial x}+Pe\frac{\partial^{2}}{\partial s_{5}^{2}z^{2}}\right)F=0,$$

where $s_{0}=\sqrt{c_{66}/c_{44}}$, $s_{5}=\sqrt{k_{11}/k_{33}}$, and $s_{k}$ (*k* = 1, 2, 3, 4) are the four roots (real positive parts) of the following algebraic equation:

(14) $$n_{0}s^{8}-n_{1}s^{6}+n_{2}s^{4}-n_{3}s^{2}+n_{4}=0.$$

### 2.3. Frequency Response Functions (FRFs)

To obtain the functions *Ψ* and *F*, the Fourier transform is performed on Equations (12) and (13), and it can be derived as

(15) $$\begin{array}{l} \tilde{\tilde{\Psi }}=A_{0}e^{-\alpha s_{0}z}+{\bar{A}}_{0}e^{\alpha s_{0}z}\\ \tilde{\tilde{F}}=\sum_{k=1}^{4}\left(A_{k}e^{-\alpha s_{k}z}+{\bar{A}}_{k}e^{\alpha s_{k}z}\right)+A_{5}e^{-rs_{5}z}+{\bar{A}}_{5}e^{rs_{5}z} \end{array}$$

in which $r=\sqrt{\alpha^{2}-imPe}$, where *i* denotes the imaginary unit; $\alpha =\sqrt{m^{2}+n^{2}}$ with the frequency variables *m* and *n* corresponding to *x* and *y* in the time domain, respectively; and the unknowns *A_k_* (*k* = 1, 2, …, 5) are determined by the specific boundary conditions.

Furthermore, solutions (in Equation (11)) for the displacement ***u***, electric potential ***ϕ***, magnetic potential ***ψ*** and temperature ***T*** in the frequency domain are given by

(16) $$\begin{array}{l} \tilde{\tilde{u}}{}^{\left(j\right)}=in\left(A_{0}^{\left(j\right)}e^{-\alpha s_{0}^{\left(j\right)}z_{j}}+{\bar{A}}_{0}^{\left(j\right)}e^{\alpha s_{0}^{\left(j\right)}z_{j}}\right)-im\sum_{k=1}^{4}\varpi_{1k}^{\left(j\right)}\left(A_{k}^{\left(j\right)}e^{-\alpha s_{k}^{\left(j\right)}z_{j}}+{\bar{A}}_{k}^{\left(j\right)}e^{\alpha s_{k}^{\left(j\right)}z_{j}}\right)\\ -im\varpi_{15}^{\left(j\right)}\left(A_{5}^{\left(j\right)}e^{-r^{\left(j\right)}s_{5}^{\left(j\right)}z_{j}}+{\bar{A}}_{5}^{\left(j\right)}e^{r^{\left(j\right)}s_{5}^{\left(j\right)}z_{j}}\right),\\ \tilde{\tilde{v}}{}^{\left(j\right)}=-im\left(A_{0}^{\left(j\right)}e^{-\alpha s_{0}^{\left(j\right)}z_{j}}+{\bar{A}}_{0}^{\left(j\right)}e^{\alpha s_{0}^{\left(j\right)}z_{j}}\right)-in\sum_{k=1}^{4}\varpi_{1k}^{\left(j\right)}\left(A_{k}^{\left(j\right)}e^{-\alpha s_{k}^{\left(j\right)}z_{j}}+{\bar{A}}_{k}^{\left(j\right)}e^{\alpha s_{k}^{\left(j\right)}z_{j}}\right)\\ -in\varpi_{15}^{\left(j\right)}\left(A_{5}^{\left(j\right)}e^{-r^{\left(j\right)}s_{5}^{\left(j\right)}z_{j}}+{\bar{A}}_{5}^{\left(j\right)}e^{r^{\left(j\right)}s_{5}^{\left(j\right)}z_{j}}\right),\\ \tilde{\tilde{w}}{}^{\left(j\right)}=-\sum_{k=1}^{4}\alpha s_{k}^{\left(j\right)}\varpi_{2k}^{\left(j\right)}\left(A_{k}^{\left(j\right)}e^{-\alpha s_{k}^{\left(j\right)}z_{j}}-{\bar{A}}_{k}^{\left(j\right)}e^{\alpha s_{k}^{\left(j\right)}z_{j}}\right)-r^{\left(j\right)}s_{5}^{\left(j\right)}\varpi_{25}^{\left(j\right)}\left(A_{5}^{\left(j\right)}e^{-r^{\left(j\right)}s_{5}^{\left(j\right)}z_{j}}-{\bar{A}}_{5}^{\left(j\right)}e^{r^{\left(j\right)}s_{5}^{\left(j\right)}z_{j}}\right),\\ \tilde{\tilde{\phi }}{}^{\left(j\right)}=-\sum_{k=1}^{4}\alpha s_{k}^{\left(j\right)}\varpi_{3k}^{\left(j\right)}\left(A_{k}^{\left(j\right)}e^{-\alpha s_{k}^{\left(j\right)}z_{j}}-{\bar{A}}_{k}^{\left(j\right)}e^{\alpha s_{k}^{\left(j\right)}z_{j}}\right)-r^{\left(j\right)}s_{5}^{\left(j\right)}\varpi_{35}^{\left(j\right)}\left(A_{5}^{\left(j\right)}e^{-r^{\left(j\right)}s_{5}^{\left(j\right)}z_{j}}-{\bar{A}}_{5}^{\left(j\right)}e^{r^{\left(j\right)}s_{5}^{\left(j\right)}z_{j}}\right),\\ \tilde{\tilde{\varphi }}{}^{\left(j\right)}=-\sum_{k=1}^{4}\alpha s_{k}^{\left(j\right)}\varpi_{4k}^{\left(j\right)}\left(A_{k}^{\left(j\right)}e^{-\alpha s_{k}^{\left(j\right)}z_{j}}-{\bar{A}}_{k}^{\left(j\right)}e^{\alpha s_{k}^{\left(j\right)}z_{j}}\right)-r^{\left(j\right)}s_{5}^{\left(j\right)}\varpi_{45}^{\left(j\right)}\left(A_{5}^{\left(j\right)}e^{-r^{\left(j\right)}s_{5}^{\left(j\right)}z_{j}}-{\bar{A}}_{5}^{\left(j\right)}e^{r^{\left(j\right)}s_{5}^{\left(j\right)}z_{j}}\right),\\ \tilde{\tilde{T}}{}^{\left(j\right)}=\varpi_{55}^{\left(j\right)}\left(A_{5}^{\left(j\right)}e^{-r^{\left(j\right)}s_{5}^{\left(j\right)}z_{j}}+{\bar{A}}_{5}^{\left(j\right)}e^{r^{\left(j\right)}s_{5}^{\left(j\right)}z_{j}}\right), \end{array}$$

where *j* represents the coating (*j* = 1) and the substrate (*j* = 2), ‘≈’ denotes the double Fourier transform operation, and the coefficients $\varpi_{1k},\ldots ,\varpi_{5k}$ (*k* = 1, 2, …, 5) are listed in Appendix A.

After performing the Fourier transform on the constitutive relations in Equation (1), and substituting Equation (15) into Equation (1), general solutions in the frequency domain of the mechanical stresses *σ_ij_*, the electric displacement *D_i_* and the magnetic induction *B_i_* are obtained:

(17) $$\begin{array}{l} \tilde{\tilde{\sigma }}{}_{xx}^{\left(j\right)}=-2c_{66}^{\left(j\right)}mn\left(A_{0}^{\left(j\right)}e^{-\alpha s_{0}^{\left(j\right)}z_{j}}+{\bar{A}}_{0}^{\left(j\right)}e^{\alpha s_{0}^{\left(j\right)}z_{j}}\right)\\ +\sum_{k=1}^{4}\left[\left(m^{2}c_{11}^{\left(j\right)}+n^{2}c_{12}^{\left(j\right)}\right)\varpi_{1k}^{\left(j\right)}+\kappa_{1k}^{\left(j\right)}\right]\left(A_{k}^{\left(j\right)}e^{-\alpha s_{k}^{\left(j\right)}z_{j}}+{\bar{A}}_{k}^{\left(j\right)}e^{\alpha s_{k}^{\left(j\right)}z_{j}}\right)\\ +\left[\left(m^{2}c_{11}^{\left(j\right)}+n^{2}c_{12}^{\left(j\right)}\right)\varpi_{15}^{\left(j\right)}+\kappa_{15}^{\left(j\right)}-\beta_{1}^{\left(j\right)}\varpi_{55}^{\left(j\right)}\right]\left(A_{5}^{\left(j\right)}e^{-r^{\left(j\right)}s_{5}^{\left(j\right)}z_{j}}+{\bar{A}}_{5}^{\left(j\right)}e^{r^{\left(j\right)}s_{5}^{\left(j\right)}z_{j}}\right),\\ \tilde{\tilde{\sigma }}{}_{yy}^{\left(j\right)}=2c_{66}^{\left(j\right)}mn\left(A_{0}^{\left(j\right)}e^{-\alpha s_{0}^{\left(j\right)}z_{j}}+{\bar{A}}_{0}^{\left(j\right)}e^{\alpha s_{0}^{\left(j\right)}z_{j}}\right)\\ +\sum_{k=1}^{4}\left[\left(m^{2}c_{12}^{\left(j\right)}+n^{2}c_{11}^{\left(j\right)}\right)\varpi_{1k}^{\left(j\right)}+\kappa_{1k}^{\left(j\right)}\right]\left(A_{k}^{\left(j\right)}e^{-\alpha s_{k}^{\left(j\right)}z_{j}}+{\bar{A}}_{k}^{\left(j\right)}e^{\alpha s_{k}^{\left(j\right)}z_{j}}\right)\\ +\left[\left(m^{2}c_{12}^{\left(j\right)}+n^{2}c_{11}^{\left(j\right)}\right)\varpi_{15}^{\left(j\right)}+\kappa_{15}^{\left(j\right)}-\beta_{1}^{\left(j\right)}\varpi_{55}^{\left(j\right)}\right]\left(A_{5}^{\left(j\right)}e^{-r^{\left(j\right)}s_{5}^{\left(j\right)}z_{j}}+{\bar{A}}_{5}^{\left(j\right)}e^{r^{\left(j\right)}s_{5}^{\left(j\right)}z_{j}}\right),\\ \tilde{\tilde{\sigma }}{}_{zz}^{\left(j\right)}=\sum_{k=1}^{4}\kappa_{2k}^{\left(j\right)}\left(A_{k}^{\left(j\right)}e^{-\alpha s_{k}^{\left(j\right)}z_{j}}+{\bar{A}}_{k}^{\left(j\right)}e^{\alpha s_{k}^{\left(j\right)}z_{j}}\right)+\left(\kappa_{25}^{\left(j\right)}-\beta_{3}^{\left(j\right)}\varpi_{55}^{\left(j\right)}\right)\left(A_{5}^{\left(j\right)}e^{-r^{\left(j\right)}s_{5}^{\left(j\right)}z_{j}}+{\bar{A}}_{5}^{\left(j\right)}e^{r^{\left(j\right)}s_{5}^{\left(j\right)}z_{j}}\right), \end{array}$$

(18) $$\begin{array}{l} \tilde{\tilde{\sigma }}{}_{xy}^{\left(j\right)}=-c_{66}^{\left(j\right)}\left(n^{2}-m^{2}\right)\left(A_{0}^{\left(j\right)}e^{-\alpha s_{0}^{\left(j\right)}z_{j}}+{\bar{A}}_{0}^{\left(j\right)}e^{\alpha s_{0}^{\left(j\right)}z_{j}}\right)\\ +\sum_{k=1}^{4}2c_{66}^{\left(j\right)}mn\varpi_{1k}^{\left(j\right)}\left(A_{k}^{\left(j\right)}e^{-\alpha s_{k}^{\left(j\right)}z_{j}}+{\bar{A}}_{k}^{\left(j\right)}e^{\alpha s_{k}^{\left(j\right)}z_{j}}\right)+2c_{66}^{\left(j\right)}mn\varpi_{15}^{\left(j\right)}\left(A_{5}^{\left(j\right)}e^{-r^{\left(j\right)}s_{5}^{\left(j\right)}z_{j}}+{\bar{A}}_{5}^{\left(j\right)}e^{r^{\left(j\right)}s_{5}^{\left(j\right)}z_{j}}\right),\\ \tilde{\tilde{\sigma }}{}_{zx}^{\left(j\right)}=-c_{44}^{\left(j\right)}in\alpha s_{0}^{\left(j\right)}\left(A_{0}^{\left(j\right)}e^{-\alpha s_{0}^{\left(j\right)}z_{j}}-{\bar{A}}_{0}^{\left(j\right)}e^{\alpha s_{0}^{\left(j\right)}z_{j}}\right)\\ +im\sum_{k=1}^{4}\kappa_{3k}^{\left(j\right)}\left(A_{k}^{\left(j\right)}e^{-\alpha s_{k}^{\left(j\right)}z_{j}}-{\bar{A}}_{k}^{\left(j\right)}e^{\alpha s_{k}^{\left(j\right)}z_{j}}\right)+im\kappa_{35}^{\left(j\right)}\left(A_{5}^{\left(j\right)}e^{-r^{\left(j\right)}s_{5}^{\left(j\right)}z_{j}}-{\bar{A}}_{5}^{\left(j\right)}e^{r^{\left(j\right)}s_{5}^{\left(j\right)}z_{j}}\right),\\ \tilde{\tilde{\sigma }}{}_{zy}^{\left(j\right)}=c_{44}^{\left(j\right)}im\alpha s_{0}^{\left(j\right)}\left(A_{0}^{\left(j\right)}e^{-\alpha s_{0}^{\left(j\right)}z_{j}}-{\bar{A}}_{0}^{\left(j\right)}e^{\alpha s_{0}^{\left(j\right)}z_{j}}\right)\\ +in\sum_{k=1}^{4}\kappa_{3k}^{\left(j\right)}\left(A_{k}^{\left(j\right)}e^{-\alpha s_{k}^{\left(j\right)}z_{j}}-{\bar{A}}_{k}^{\left(j\right)}e^{\alpha s_{k}^{\left(j\right)}z_{j}}\right)+in\kappa_{35}^{\left(j\right)}\left(A_{5}^{\left(j\right)}e^{-r^{\left(j\right)}s_{5}^{\left(j\right)}z_{j}}-{\bar{A}}_{5}^{\left(j\right)}e^{r^{\left(j\right)}s_{5}^{\left(j\right)}z_{j}}\right). \end{array}$$

(19) $$\begin{array}{l} \tilde{\tilde{D}}{}_{x}^{\left(j\right)}=-e_{15}^{\left(j\right)}in\alpha s_{0}^{\left(j\right)}\left(A_{0}^{\left(j\right)}e^{-\alpha s_{0}^{\left(j\right)}z_{j}}-{\bar{A}}_{0}^{\left(j\right)}e^{\alpha s_{0}^{\left(j\right)}z_{j}}\right)\\ +im\sum_{k=1}^{4}\kappa_{4k}^{\left(j\right)}\left(A_{k}^{\left(j\right)}e^{-\alpha s_{k}^{\left(j\right)}z_{j}}-{\bar{A}}_{k}^{\left(j\right)}e^{\alpha s_{k}^{\left(j\right)}z_{j}}\right)+im\kappa_{45}^{\left(j\right)}\left(A_{5}^{\left(j\right)}e^{-r^{\left(j\right)}s_{5}^{\left(j\right)}z_{j}}-{\bar{A}}_{5}^{\left(j\right)}e^{r^{\left(j\right)}s_{5}^{\left(j\right)}z_{j}}\right),\\ \tilde{\tilde{D}}{}_{y}^{\left(j\right)}=e_{15}^{\left(j\right)}im\alpha s_{0}^{\left(j\right)}\left(A_{0}^{\left(j\right)}e^{-\alpha s_{0}^{\left(j\right)}z_{j}}-{\bar{A}}_{0}^{\left(j\right)}e^{\alpha s_{0}^{\left(j\right)}z_{j}}\right)\\ +in\sum_{k=1}^{4}\kappa_{4k}^{\left(j\right)}\left(A_{k}^{\left(j\right)}e^{-\alpha s_{k}^{\left(j\right)}z_{j}}-{\bar{A}}_{k}^{\left(j\right)}e^{\alpha s_{k}^{\left(j\right)}z_{j}}\right)+in\kappa_{45}^{\left(j\right)}\left(A_{5}^{\left(j\right)}e^{-r^{\left(j\right)}s_{5}^{\left(j\right)}z_{j}}-{\bar{A}}_{5}^{\left(j\right)}e^{r^{\left(j\right)}s_{5}^{\left(j\right)}z_{j}}\right),\\ \tilde{\tilde{D}}{}_{z}^{\left(j\right)}=\sum_{k=1}^{4}\kappa_{5k}^{\left(j\right)}\left(A_{k}^{\left(j\right)}e^{-\alpha s_{k}^{\left(j\right)}z_{j}}+{\bar{A}}_{k}^{\left(j\right)}e^{\alpha s_{k}^{\left(j\right)}z_{j}}\right)+\left(\kappa_{55}^{\left(j\right)}+p_{3}^{\left(j\right)}\varpi_{55}^{\left(j\right)}\right)\left(A_{5}^{\left(j\right)}e^{-r^{\left(j\right)}s_{5}^{\left(j\right)}z_{j}}+{\bar{A}}_{5}^{\left(j\right)}e^{r^{\left(j\right)}s_{5}^{\left(j\right)}z_{j}}\right), \end{array}$$

(20) $$\begin{array}{l} \tilde{\tilde{B}}{}_{x}^{\left(j\right)}=-q_{15}^{\left(j\right)}in\alpha s_{0}^{\left(j\right)}\left(A_{0}^{\left(j\right)}e^{-\alpha s_{0}^{\left(j\right)}z_{j}}-{\bar{A}}_{0}^{\left(j\right)}e^{\alpha s_{0}^{\left(j\right)}z_{j}}\right)\\ +im\sum_{k=1}^{4}\kappa_{6k}^{\left(j\right)}\left(A_{k}^{\left(j\right)}e^{-\alpha s_{k}^{\left(j\right)}z_{j}}-{\bar{A}}_{k}^{\left(j\right)}e^{\alpha s_{k}^{\left(j\right)}z_{j}}\right)+im\kappa_{65}^{\left(j\right)}\left(A_{5}^{\left(j\right)}e^{-r^{\left(j\right)}s_{5}^{\left(j\right)}z_{j}}-{\bar{A}}_{5}^{\left(j\right)}e^{r^{\left(j\right)}s_{5}^{\left(j\right)}z_{j}}\right),\\ \tilde{\tilde{B}}{}_{y}^{\left(j\right)}=q_{15}^{\left(j\right)}im\alpha s_{0}^{\left(j\right)}\left(A_{0}^{\left(j\right)}e^{-\alpha s_{0}^{\left(j\right)}z_{j}}-{\bar{A}}_{0}^{\left(j\right)}e^{\alpha s_{0}^{\left(j\right)}z_{j}}\right)\\ +in\sum_{k=1}^{4}\kappa_{6k}^{\left(j\right)}\left(A_{k}^{\left(j\right)}e^{-\alpha s_{k}^{\left(j\right)}z_{j}}-{\bar{A}}_{k}^{\left(j\right)}e^{\alpha s_{k}^{\left(j\right)}z_{j}}\right)+in\kappa_{65}^{\left(j\right)}\left(A_{5}^{\left(j\right)}e^{-r^{\left(j\right)}s_{5}^{\left(j\right)}z_{j}}-{\bar{A}}_{5}^{\left(j\right)}e^{r^{\left(j\right)}s_{5}^{\left(j\right)}z_{j}}\right),\\ \tilde{\tilde{B}}{}_{z}^{\left(j\right)}=\sum_{k=1}^{4}\kappa_{7k}^{\left(j\right)}\left(A_{k}^{\left(j\right)}e^{-\alpha s_{k}^{\left(j\right)}z_{j}}+{\bar{A}}_{k}^{\left(j\right)}e^{\alpha s_{k}^{\left(j\right)}z_{j}}\right)+\left(\kappa_{75}^{\left(j\right)}+\lambda_{3}^{\left(j\right)}\varpi_{55}^{\left(j\right)}\right)\left(A_{5}^{\left(j\right)}e^{-r^{\left(j\right)}s_{5}^{\left(j\right)}z_{j}}+{\bar{A}}_{5}^{\left(j\right)}e^{r^{\left(j\right)}s_{5}^{\left(j\right)}z_{j}}\right), \end{array}$$

where the expressions of the shear stress, electric displacement, magnetic induction and the coefficients $\kappa_{1k},\kappa_{2k},\ldots ,\kappa_{5k}$ (*k* = 1, 2, …, 5) in Equation (16) are listed in Appendix A.

In order to solve the unknowns $A_{0},A_{1},\ldots ,A_{5}$ and ${\bar{A}}_{0},{\bar{A}}_{1},\ldots ,{\bar{A}}_{5}$, the boundary conditions of the coating surface and the interface between the coating and the substrate are employed. At the coating surface (*z*_1_ = 0), the normal pressure *p* and the heat flow *q* are applied and the boundary conditions can be prescribed as

(21) $$\begin{array}{l} {\left.\tilde{\tilde{\sigma }}{}_{zz}^{\left(1\right)}\right|}_{z_{1}=0}=-\tilde{\tilde{p}},{\left.\tilde{\tilde{\sigma }}{}_{xz}^{\left(1\right)}\right|}_{z_{1}=0}=-\mu_{f}\tilde{\tilde{p}},{\left.\tilde{\tilde{\sigma }}{}_{yz}^{\left(1\right)}\right|}_{z_{1}=0}=0,\\ {\left.\tilde{\tilde{D}}{}_{z}^{\left(1\right)}\right|}_{z_{1}=0}=-{\tilde{\tilde{q}}}_{b},{\left.\tilde{\tilde{B}}{}_{z}^{\left(1\right)}\right|}_{z_{1}=0}=-{\tilde{\tilde{g}}}_{b},{\left.k_{33}^{\left(1\right)}\frac{\partial }{\partial z}\tilde{\tilde{T}}{}^{\left(1\right)}\right|}_{z_{1}=0}=-\tilde{\tilde{q}}. \end{array}$$

In the present study, the displacement and the stress across the interfaces between the coating and the substrate are regarded as continuously transmitted; therefore, the stresses, displacement, electric potential, magnetic potential, electric displacements and magnetic induction should be transmitted continuously at the interface between the coating and the substrate as

(22) $$\begin{array}{l} {\left.\tilde{\tilde{\sigma }}{}_{zz}^{\left(1\right)}\right|}_{z_{1}=h_{1}}={\left.\tilde{\tilde{\sigma }}{}_{zz}^{\left(2\right)}\right|}_{z_{2}=0},{\left.\tilde{\tilde{\sigma }}{}_{xz}^{\left(1\right)}\right|}_{z_{1}=h_{1}}={\left.\tilde{\tilde{\sigma }}{}_{xz}^{\left(2\right)}\right|}_{z_{2}=0},\\ {\left.\tilde{\tilde{\sigma }}{}_{yz}^{\left(1\right)}\right|}_{z_{1}=h_{1}}={\left.\tilde{\tilde{\sigma }}{}_{yz}^{\left(2\right)}\right|}_{z_{2}=0},{\left.\tilde{\tilde{u}}{}_{zz}^{\left(1\right)}\right|}_{z_{1}=h_{1}}={\left.\tilde{\tilde{u}}{}_{zz}^{\left(2\right)}\right|}_{z_{2}=0},\\ {\left.\tilde{\tilde{u}}{}_{xz}^{\left(1\right)}\right|}_{z_{1}=h_{1}}={\left.\tilde{\tilde{u}}{}_{xz}^{\left(2\right)}\right|}_{z_{2}=0},{\left.\tilde{\tilde{u}}{}_{yz}^{\left(1\right)}\right|}_{z_{1}=h_{1}}={\left.\tilde{\tilde{u}}{}_{yz}^{\left(2\right)}\right|}_{z_{2}=0},\\ {\left.\tilde{\tilde{\phi }}{}_{z}^{\left(1\right)}\right|}_{z_{1}=h_{1}}={\left.\tilde{\tilde{\phi }}{}_{z}^{\left(2\right)}\right|}_{z_{2}=0},{\left.\tilde{\tilde{\varphi }}{}_{z}^{\left(1\right)}\right|}_{z_{1}=h_{1}}={\left.\tilde{\tilde{\varphi }}{}_{z}^{\left(2\right)}\right|}_{z_{2}=0},\\ {\left.\tilde{\tilde{T}}{}^{\left(1\right)}\right|}_{z_{1}=h_{1}}={\left.\tilde{\tilde{T}}{}^{\left(2\right)}\right|}_{z_{2}=0},{\left.\tilde{\tilde{D}}{}_{z}^{\left(1\right)}\right|}_{z_{1}=h_{1}}={\left.\tilde{\tilde{D}}{}_{z}^{\left(2\right)}\right|}_{z_{2}=0},\\ {\left.\tilde{\tilde{B}}{}_{z}^{\left(1\right)}\right|}_{z_{1}=h_{1}}={\left.\tilde{\tilde{B}}{}_{z}^{\left(2\right)}\right|}_{z_{2}=0},{\left.k_{33}^{\left(1\right)}\frac{\partial }{\partial z}\tilde{\tilde{T}}{}^{\left(1\right)}\right|}_{z_{1}=h_{1}}={\left.k_{33}^{\left(2\right)}\frac{\partial }{\partial z}\tilde{\tilde{T}}{}^{\left(2\right)}\right|}_{z_{2}=0}. \end{array}$$

In addition, for the infinite half-space, the stress, displacement, electric displacement, magnetic induction and temperature are treated as zero at infinity ($z_{2}\to \infty$), which can be described as

(23) $$\begin{array}{l} {\left.\tilde{\tilde{\sigma }}{}_{ij}^{\left(2\right)}\right|}_{z_{2}\to \infty }=0,\;{\left.\tilde{\tilde{u}}{}_{i}^{\left(2\right)}\right|}_{z_{2}\to \infty }=0,\;{\left.\tilde{\tilde{D}}{}_{i}^{\left(2\right)}\right|}_{z_{2}\to \infty }=0,\;{\left.\tilde{\tilde{B}}{}_{i}^{\left(2\right)}\right|}_{z_{2}\to \infty }=0,\\ {\left.\tilde{\tilde{\phi }}{}^{\left(2\right)}\right|}_{z_{2}\to \infty }=0,\;{\left.\tilde{\tilde{\varphi }}{}^{\left(2\right)}\right|}_{z_{2}\to \infty }=0,\;{\left.\tilde{\tilde{T}}{}^{\left(2\right)}\right|}_{z_{2}\to \infty }=0,\; \end{array}$$

and $\bar{A}{}_{k}^{\left(2\right)}=0\left(k=1,\ldots ,5\right)$ can be obtained by substituting Equations (16)–(20) into the boundary conditions (Equation (23)). Therefore, based on the boundary conditions in Equations (21) and (22), the eighteen unknown coefficients for the one-layered MEE material can be determined by the following steps.

Firstly, we substitute a general solution of the temperature in the frequency domain (Equation (15)) to the boundary condition that relates to the temperature. They can be written in matrix form as follows:

(24) $$\left[\begin{array}{ccc} 1 & -1 & 0\\ \frac{\varpi_{55}^{\left(1\right)}\theta_{5}}{\varpi_{55}^{\left(2\right)}} & \frac{\varpi_{55}^{\left(1\right)}}{\varpi_{55}^{\left(2\right)}\theta_{5}} & -1\\ \chi_{2}\theta_{5} & -\chi_{2}/\theta_{5} & -1 \end{array}\right]\left[\begin{array}{c} A_{5}^{\left(1\right)}\\ \bar{A}{}_{5}^{\left(1\right)}\\ A_{5}^{\left(2\right)} \end{array}\right]=\left[\begin{array}{c} \frac{\tilde{\tilde{q}}}{k_{33}^{\left(1\right)}r_{1}s_{5}^{\left(1\right)}\varpi_{55}^{\left(1\right)}}\\ 0\\ 0 \end{array}\right]$$

(25) $$\chi_{2}=\frac{k_{33}^{\left(1\right)}r_{1}s_{5}^{\left(1\right)}\varpi_{55}^{\left(1\right)}}{k_{33}^{\left(2\right)}r_{2}s_{5}^{\left(2\right)}\varpi_{55}^{\left(2\right)}},\theta_{5}=e^{-r_{1}s_{5}^{\left(1\right)}h_{1}}$$

Equation (24) only contains the unknown coefficients $A_{5}^{\left(1\right)}$, $\bar{A}{}_{5}^{\left(1\right)}$, and $A_{5}^{\left(2\right)}$, which can be obtained independently.

(26) $$\begin{array}{l} A_{5}^{\left(1\right)}=\frac{\tilde{\tilde{q}}\left(\varpi_{55}^{\left(1\right)}+\varpi_{55}^{\left(2\right)}\chi_{2}\right)}{k_{33}^{\left(1\right)}r_{1}s_{5}^{\left(1\right)}\varpi_{55}^{\left(1\right)}\left[\varpi_{55}^{\left(1\right)}+\varpi_{55}^{\left(2\right)}\chi_{2}+\left(\varpi_{55}^{\left(1\right)}-\varpi_{55}^{\left(2\right)}\chi_{2}\right)\theta_{5}^{2}\right]}\\ \bar{A}{}_{5}^{\left(1\right)}=-\frac{\left(\varpi_{55}^{\left(1\right)}-\varpi_{55}^{\left(2\right)}\chi_{2}\right)\theta_{5}^{2}}{\varpi_{55}^{\left(1\right)}+\varpi_{55}^{\left(2\right)}\chi_{2}}A_{5}^{\left(1\right)}\\ A_{5}^{\left(2\right)}=\chi_{2}\theta_{5}A_{5}^{\left(1\right)}-\frac{\chi_{2}}{\theta_{5}}A_{5}^{\left(1\right)} \end{array}.$$

Then, by performing some operations, undetermined coefficients $A_{k}^{\left(j\right)}$ and $\bar{A}{}_{k}^{\left(j\right)}\left(k=1,\ldots ,5\right)$ in the equations can be eliminated, and only $A_{0}^{\left(1\right)}$, $A_{0}^{\left(2\right)}$, and $\bar{A}{}_{0}^{\left(1\right)}$ are left in the following equations:

(27) $$\left[\begin{array}{ccc} 1 & -1 & 0\\ \theta_{0} & 1/\theta_{0} & -1\\ \chi_{1}\theta_{0} & -\chi_{1}/\theta_{0} & -1 \end{array}\right]\left[\begin{array}{c} A_{0}^{\left(1\right)}\\ \bar{A}{}_{0}^{\left(1\right)}\\ A_{0}^{\left(2\right)} \end{array}\right]=\left[\begin{array}{c} \frac{-in\mu_{f}\tilde{\tilde{p}}}{c_{44}^{\left(1\right)}\alpha^{3}s_{0}^{\left(1\right)}}\\ 0\\ 0 \end{array}\right],$$

(28) $$\chi_{1}=\frac{c_{44}^{\left(1\right)}s_{0}^{\left(1\right)}}{c_{44}^{\left(2\right)}s_{0}^{\left(2\right)}},\theta_{0}=e^{-\alpha s_{0}^{\left(1\right)}h_{1}}.$$

By solving Equation (27), the expressions of $A_{0}^{\left(1\right)}$, $A_{0}^{\left(2\right)}$, and $\bar{A}{}_{0}^{\left(1\right)}$ can be obtained:

(29) $$\begin{array}{l} A_{0}^{\left(1\right)}=\frac{-in\mu_{f}\tilde{\tilde{p}}\left(1+\chi_{1}\right)}{c_{44}^{\left(1\right)}\alpha^{3}s_{0}^{\left(1\right)}\left[\left(1+\chi_{1}\right)+\left(1-\chi_{1}\right)\theta_{0}^{2}\right]}\\ \bar{A}{}_{0}^{\left(1\right)}=-\frac{\left(1-\chi_{1}\right)\theta_{0}^{2}}{\left(1+\chi_{1}\right)}A_{0}^{\left(1\right)}\\ A_{0}^{\left(2\right)}=\chi_{1}\theta_{0}A_{0}^{\left(1\right)}-\frac{\chi_{1}}{\theta_{0}}\bar{A}{}_{0}^{\left(1\right)} \end{array}$$

Thus far, the unknowns are reduced from eighteen to twelve. Similar to the above, to solve $A_{0}^{\left(1\right)}$, $A_{0}^{\left(2\right)}$, and $\bar{A}{}_{0}^{\left(1\right)}$, a set of equations that only include $A_{k}^{\left(j\right)}$ and $\bar{A}{}_{k}^{\left(j\right)}\left(k=1,\ldots ,4\right)$ can be obtained. Combining other equations in the boundary conditions to calculate $A_{k}^{\left(j\right)}$ and $\bar{A}{}_{k}^{\left(j\right)}\left(k=1,\ldots ,4\right)$, the matrix can be written as

(30) $$\left[\begin{array}{ccc} N_{1k}^{\left(1\right)} & UN_{1k}^{\left(1\right)} & 0\\ N_{1k}^{\left(1\right)}\theta_{k}^{\left(1\right)} & UN_{1k}^{\left(1\right)}/\theta_{k}^{\left(1\right)} & N_{1k}^{\left(2\right)}\\ N_{2k}^{\left(1\right)}\theta_{k}^{\left(1\right)} & -UN_{2k}^{\left(1\right)}/\theta_{k}^{\left(1\right)} & N_{2k}^{\left(2\right)} \end{array}\right]\left[\begin{array}{c} A_{k}^{\left(1\right)}\\ \bar{A}{}_{k}^{\left(1\right)}\\ A_{k}^{\left(2\right)} \end{array}\right]=\left[\begin{array}{c} W_{1}\\ W_{2}\\ W_{3} \end{array}\right]$$

where the submatrices *U*, $N_{1k}^{\left(j\right)}$, $N_{2k}^{\left(j\right)}$, *W*_0_, *W*_1_, and *W*_2_ are listed in Appendix B (the expressions of $A_{5}^{\left(1\right)}$, $\bar{A}{}_{5}^{\left(1\right)}$, and $A_{5}^{\left(2\right)}$ contained in *W*_0_, *W*_1_, and *W*_2_ are shown in Equation (26)). $A_{k}^{\left(j\right)}={\left[\begin{array}{cccc} A_{1}^{\left(j\right)} & A_{2}^{\left(j\right)} & A_{3}^{\left(j\right)} & A_{4}^{\left(j\right)} \end{array}\right]}^{T}\left(j=1,2\right)$ and $\bar{A}{}_{k}^{\left(1\right)}={\left[\begin{array}{cccc} \bar{A}{}_{1}^{\left(1\right)} & \bar{A}{}_{2}^{\left(1\right)} & \bar{A}{}_{3}^{\left(1\right)} & \bar{A}{}_{4}^{\left(1\right)} \end{array}\right]}^{T}$ are the submatrices of the undetermined coefficients. Hence, the remaining undetermined coefficients can be obtained by solving the linear equations in Equation (30).

The FRFs of the general solutions for the magneto-electro-thermo-elastic field of the layered material considering transverse isotropy have been obtained. Using this solution, the mechanical stresses, displacement, electric potential, magnetic potential and temperature rise can be obtained for a given load. The advantage of the general solution is that the FRFs can be determined efficiently based on the fast Fourier transform algorithm, and it is an elementary solution for a unit load that can be used for a distributed load by summarizing the effects from all loading units. The disadvantage of the general solution is that the interfacial defects and the inhomogeneity of the coating–substrate system are not considered. The general solution obtained in this section plays an important role in the semi-analytical model. For example, in the contact model, the surface displacement is necessary to calculate the contact equilibrium, and the thermal contact response (mechanical stresses, displacement, electric potential, magnetic potential and temperature rise) under a contact load is finally obtained by the general solution.

## 3. Semi-Analytical Model for Thermal Contact Problem

In the contact model, as shown in Figure 1, the elastic contact problem in the vertical direction between the sliding ball and the half-space can be described with the following system of equations and inequalities [34]:

(31) $$\begin{array}{l} \int_{A_{c}}^{}p\left(x,y\right)dxdy=P,\\ g\left(x,y\right)=0,\;p\left(x,y\right)>0\Rightarrow \forall \left(x,y\right)\in A_{c},\\ g\left(x,y\right)>0,\;p\left(x,y\right)=0\Rightarrow \forall \left(x,y\right)\notin A_{c}, \end{array}$$

where *A_c_* is the contact area, *p*(*x*, *y*) is the vertical pressure in the *z* direction within the contact area, *P* represents the normal load acting on the ball, and *g*(*x*, *y*) denotes the gap between the two contact bodies. Zhang et al. [35] further introduced the effect of the surface charge and magnetic charge during contact processes, namely

(32) $$\begin{array}{l} \int_{A_{c}}^{}q_{b}dxdy=Q_{b},\\ \int_{A_{c}}^{}g_{b}dxdy=G_{b} \end{array}$$

where *q_b_* and *g_b_* denote the surface electric and magnetic charge densities, respectively; *Q_b_* and *G_b_* are the surface total electric and magnetic charges. Note that the electric and the magnetic charges are assumed to be uniformly distributed on the surface of the half-space.

Sliding contact usually results in frictional heat generation, where all of the work generated by friction resulting from the sliding of the contact ball is ideally converted into heat, and the total heat flux in the contact area can be determined by *q* = *pμ_f_v*_s_. Based on the hypothesis of an equal temperature on the surfaces between the two contact bodies [36], the total heat flux can be partitioned by the sliding ball and the half-space as follows:

(33) $$\begin{array}{l} \tilde{\tilde{q}}={\tilde{\tilde{q}}}_{1}+{\tilde{\tilde{q}}}_{2},\\ \left(\hat{C}{}_{1}^{q}+\hat{C}{}_{2}^{q}\right){\tilde{\tilde{q}}}_{1}=\hat{C}{}_{2}^{q}{\tilde{\tilde{q}}}_{2}, \end{array}$$

where *q*_1_ and *q*_2_ are the heat fluxes flowing into the ball and the half-space; $\hat{C}{}_{1}^{q}$ and $\hat{C}{}_{2}^{q}$ are the influence coefficient matrices of the temperature rise of the two contact bodies.

The surface gap *g* between the two contact bodies in Equation (31) includes the initial vertical gap *g*_0_, the relative rigid approach *δ* and the surface normal displacement caused by multiple loads that have the form of

(34) $$g=g_{0}+u_{z}^{p}+u_{z}^{q}\left(x,y\right)+u_{z}^{q_{b}}\left(x,y\right)+u_{z}^{q_{g}}\left(x,y\right)-\delta ,$$

where $u_{z}^{p}$, $u_{z}^{q}$, $u_{z}^{p_{b}}$, and $u_{z}^{p_{b}}$ are the surface normal displacement caused by the surface pressure, the heat flux and the electric and the magnetic charges. Furthermore, the contact equilibrium equation (Equation (31)) and the heat partition equation (Equation (33)) can be solved via the conjugate gradient method (CGM) [34]. The whole numerical thermal contact analysis procedure of the MEE material should include the following steps.

(1) Parameter initialization. The material parameters, including the elastic, electric, magnetic, and thermal parameters; the surface topography of the contact ball and the half-space, the multiple loads (normal force, sliding velocity, surface electric and magnetic charges); the calculation area; and the mesh size, need to be determined.

(2) Contact pressure calculation. CGM is adopted to solve the contact equilibrium equation (Equation (31)), thus obtaining the surface contact pressure *p*(*x*, *y*) with the effects of a normal load, and the surface electric and magnetic charges. The surface tangential force can be obtained by *p_x_*(*x*, *y*) = *μ_f_ p*(*x*, *y*).

(3) Surface heat flux calculation. The total heat flux can be evaluated by *q*(*x*, *y*) = *μ_f_ p*(*x*, *y*) *v_s_* and further divided into the two contact bodies by Equation (33). With the aid of CGM, the surface heat fluxes *q*_1_ and *q*_2_ can be determined.

(4) Surface topography update. The surface displacements caused by multiple loads ($u_{z}^{p}$, $u_{z}^{q}$, $u_{z}^{p_{b}}$ and $u_{z}^{p_{b}}$) are calculated by the DC-FFT algorithm to address the gap in Equation (34), which is constantly updated by looping steps (3) and (4) until the multiple surface loads converge.

(5) Results calculation. The temperature rise, stress, electric potential and magnetic potential can be obtained by the DC-FFT algorithm with ICs. The specific implementation can be found in Ref [37].

## 4. Results and Discussion

A particular multi-ferroic composite material, BaTiO_3_-CoFe_2_O_4_, is selected, whose material constants are given in Ref. [38]. Note that the volumetric specific heat *c_h_* is obtained using the method described in Ref. [39], with a volume fraction of 50% for BaTiO_3_ and CoFe_2_O_4._ The substrate is composed of multi-ferroic composite material BaTiO_3_-CoFe_2_O_4_ for all simulations in this section, unless otherwise indicated. The radius of the loaded sliding ball is 50 mm, and the material constants are the same as in the substrate. The coating material constants and thickness are set according to different needs. In addition, the maximum Hertzian contact radius *r*, pressure *p*_0_ for transversely isotropic contact, equivalent electric potential *ϕ*_0_, equivalent magnetic potential *φ*_0_, and maximum surface temperature *T*_0_ are used to normalize the numerical results, which can be calculated as [33,35]

(35) $$a_{0}=0.9086\sqrt[{3}]{\sum_{l=1}^{2}\zeta_{l}PR_{b}}$$

(36) $$p_{h}=0.5784\sqrt[{3}]{\frac{P}{{\left(\sum_{l=1}^{2}\zeta_{l}\right)}^{2}R_{b}^{2}}}$$

(37) $$\phi_{0}=p_{0}a_{0}/e_{33},\varphi_{0}=p_{0}a_{0}/q_{33},T_{0}=p_{0}\mu_{f}v_{0}a_{0}/k_{33}$$

(38) $$\zeta_{l}=\frac{1}{2}{\left[\frac{\left(s_{1}+s_{2}\right)c_{11}}{s_{1}s_{2}\left(c_{11}c_{33}-c_{13}^{2}\right)}\right]}_{l},l=1,2$$

where *P* is the normal load, *R*_b_ is the radius of the elastic ball, *v*_0_ = 1 m/s is the sliding speed, and *μ_f_* = 0.2 is the friction coefficient. Subscript *l* represents the ball (*l* = 1) and the half-space (*l* = 2), respectively.

### 4.1. Model Verification

In order to verify the effectiveness of the thermal contact modeling of the transversely isotropic MEE coating, comparative analyses are carried out by using the proposed model and FEM (provided by ABAQUS v2017). Note that for the existing commercial FEM software, there is no complete module to conduct the magneto-electro-thermo-elastic simulation. Therefore, comparative studies for piezoelectric and thermoelastic cases obtained with the degenerate solution of the proposed method and the FEM are implemented. Here, the coated material surface is subjected to an assumed Hertzian-type load *p*(*x*, *y*) (piezoelectric case) or a heat flux *q*(*x*, *y*) (thermoelastic case) as follows:

(39) $$\begin{array}{l} p\left(x,y\right)=\sqrt{1-\frac{x^{2}}{r^{2}}-\frac{y^{2}}{r^{2}}},\\ q\left(x,y\right)=1000\sqrt{1-\frac{x^{2}}{r^{2}}-\frac{y^{2}}{r^{2}}}, \end{array}$$

where the radius of the load distribution *r* is set to be 1, and the coating thickness *h* = 0.5 *r*. Different coatings characterized by varying elastic constants *c_ij_* and heat conductivities *k_ij_* are employed within the contexts of the piezoelectric and thermoelastic cases, and other material properties can be found in Ref. [38]. In addition, in the piezoelectric case, except for the elastic constants *c_ij_* and the electric constants *e_ij_*, *ε_ij_*, the remaining parameters are set to be zero; two types of coatings are designed, namely a soft coating ($c_{ij}^{\left(1\right)}/c_{ij}^{\left(2\right)}=0.5$) and hard coating ($c_{ij}^{\left(1\right)}/c_{ij}^{\left(2\right)}=2$), for the piezoelectric case, while the rest of the coating parameters are the same as for the substrate. In the thermoelastic case, except for the elastic and thermal constants *c_ij_*, *k_ij_*, and *β_i_*, the remaining parameters are set to be zero; two types of coatings of different thermal conductivities, $k_{ij}^{\left(1\right)}/k_{ij}^{\left(2\right)}=2$ and $k_{ij}^{\left(1\right)}/k_{ij}^{\left(2\right)}=0.5$, are also designed, while the remining coating parameters are identical to those of the substrate.

**Figure 2:**
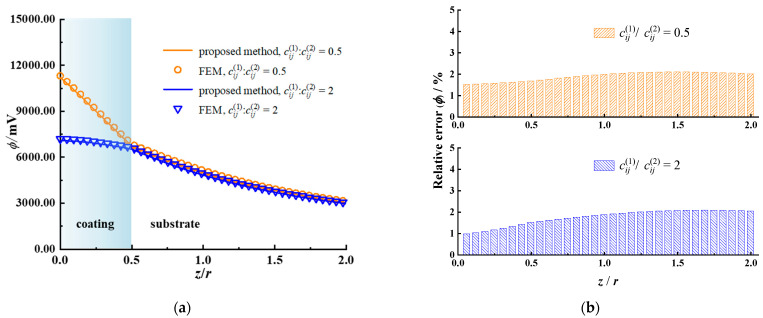

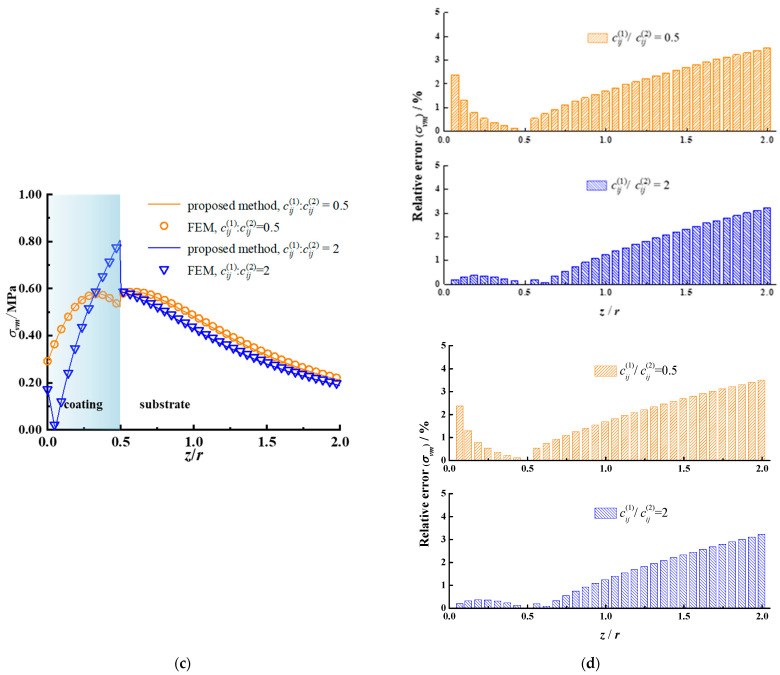
Subsurface electric potential, von Mises stress, and their relative error for different coating materials obtained with the proposed model and FEM for piezoelectric case. (**a**) Electric potential, (**b**) relative error, electric potential, (**c**) von Mises stress along the *z* axis, (**d**) relative error, von Mises stress.

**Figure 3:**
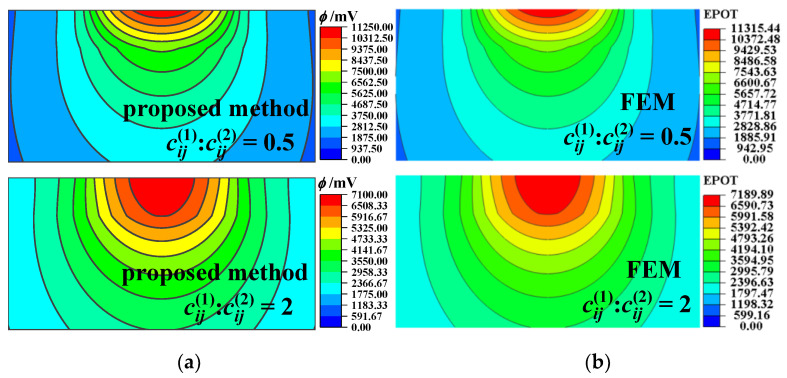

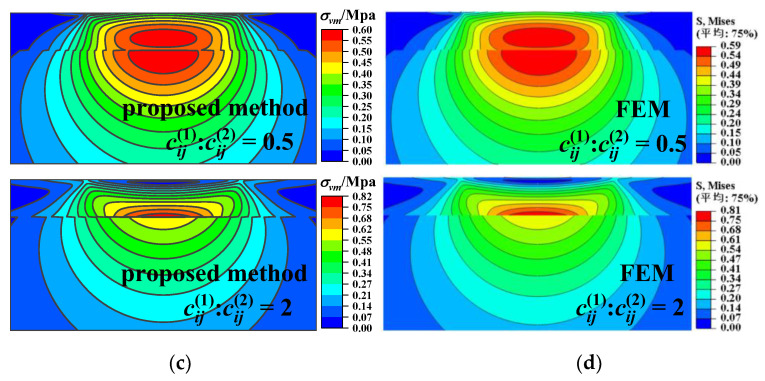
Electric potential and von Mises stress in the *x–z* plane for different coating materials for piezoelectric case. (**a**) Electric potential obtained by the proposed model, (**b**) electric potential obtained by the FEM, (**c**) von Mises stress obtained by the proposed model, (**d**) von Mises stress obtained by the FEM (the non-English words in the figure mean “average”).

**Figure 4:**
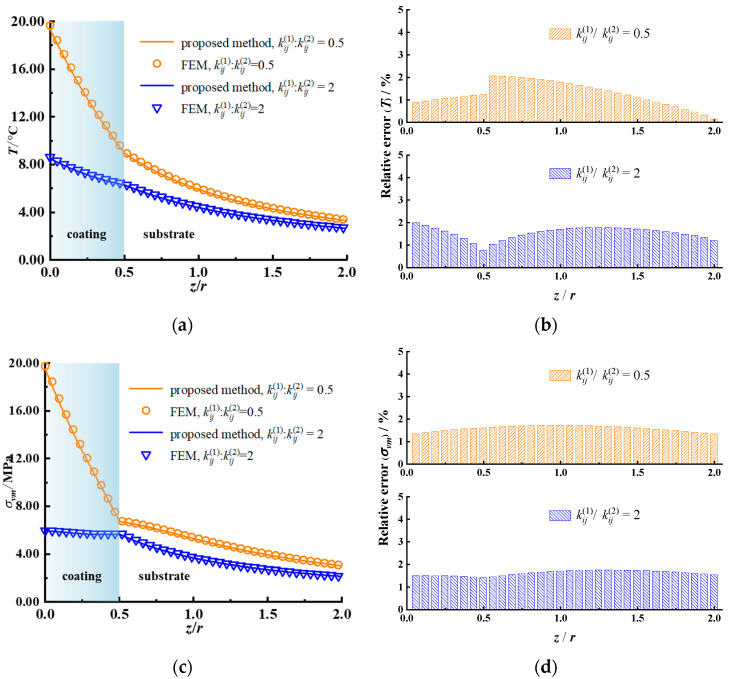
Subsurface temperature rise, von Mises stress, and their relative error for different coating materials obtained with the proposed method and FEM for the thermoelastic case. (**a**) Temperature rise along the *z* axis, (**b**) relative error, temperature rise, (**c**) von Mises stress along the *z* axis, (**d**) relative error, von Mises stress.

**Figure 5:**
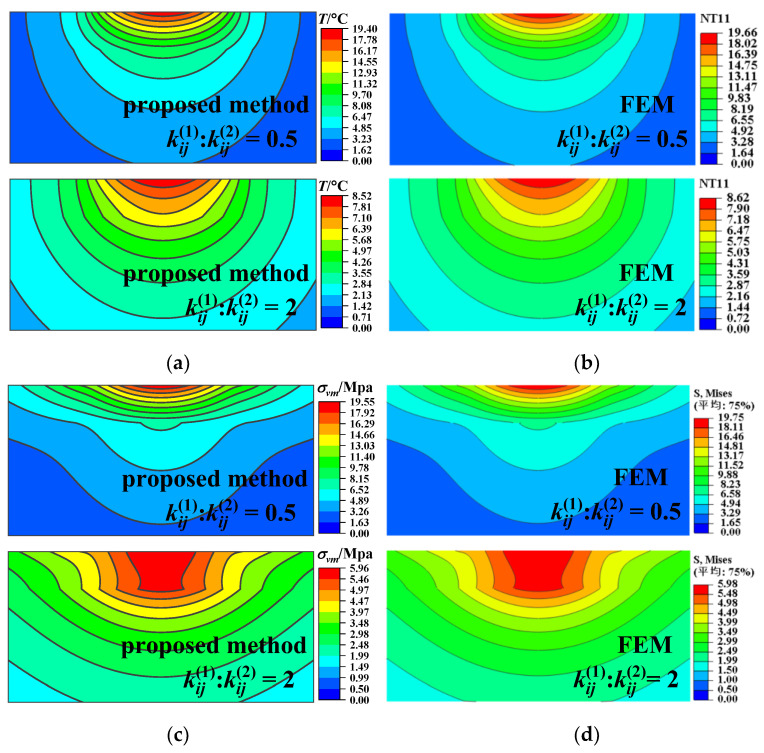
Temperature rise and von Mises stress in the *x–z* plane for different coating materials for the thermoelastic case. (**a**) Temperature rise obtained by the proposed model, (**b**) temperature rise obtained by the FEM, (**c**) von Mises stress obtained by the proposed model, (**d**) von Mises stress obtained by the FEM (the non-English words in the figure mean “average”).

The whole calculation domain is chosen as 4 *r* × 4 *r* × 2 *r* and meshed into 128 × 128 × 256 cuboidal elements sharing an identical size. Accordingly, a corresponding example is given via the axisymmetric model of FEM as a benchmark. A larger calculation domain is selected as 30 *r* × 30 *r* × 30 *r* to simulate the half-space substrate accurately. At the bottom, the displacement and the potential/temperature are set to zero. The number of discretized quad-dominated piezoelectric/temperature–displacement elements is 88,020. The calculated results for the piezoelectric case and the thermoelastic case are illustrated in Figure 2, Figure 3, Figure 4 and Figure 5, respectively.

Figure 2 exhibits the obtained electric potential, the von Mises stress along the *z* axis, and their relative error utilizing the proposed model and the FEM. The relative error is defined as the ratio of the absolute difference in the values obtained with the two methods to the value obtained by the proposed method. In the coating, the electric potential is higher in the case with a soft coating ($c_{ij}^{\left(1\right)}/c_{ij}^{\left(2\right)}=0.5$) than that with a hard coating ($c_{ij}^{\left(1\right)}/c_{ij}^{\left(2\right)}=2$). The von Mises stress has a noticeable difference at the boundary between the coating and substrate, and its value in the case with a hard coating fluctuates more significantly than that with a soft coating. The reason for this phenomenon is that the material dissimilarity between the coating and the substrate leads to stress jumping at the interface. It is known that stress is the product of the elastic constants and strain. For the interface belonging to both the coating and the substrate, the strain is the same, while the stress is different due to the disparate elastic constants. The relative error for the electric potential and the von Mises stress obtained with the two methods is less than 3%, demonstrating the good accuracy of the proposed method. Both the electric potential and von Mises stress within the substrate are slightly affected by the coating material’s properties.

Figure 3 shows the calculation results of the electric potential and the von Mises stress in the *x*–*z* plane. When the coating material is softer than the substrate ($c_{ij}^{\left(1\right)}/c_{ij}^{\left(2\right)}=0.5$), the electric potential is more concentrated near the surface, while the stress is more concentrated near the interface in the hard coating case. The distribution of the von Mises stress is discontinuous between the coating and the substrate, and the maximum stress occurs at the interface of the hard coating side.

The temperature rise, the von Mises stress along the *z* axis, and their relative error for the coatings of different heat conductivity are shown in Figure 4. Lower coating heat conductivity leads to a larger temperature rise near the surface. In the coating (*z* < *h*), the thermal stress caused by heat flux in the case of low heat conductivity is greater than that in the case of high heat conductivity. The relative error of the temperature rise and the von Mises stress obtained with the two methods is less than 2%, providing a verification of the good accuracy of the proposed method.

Figure 5 depicts the temperature rise and the von Mises stress contours for different coating thermal conductivities. The main stress concentration region in the case of low coating thermal conductivity is closer to the surface than that in the case of high coating thermal conductivity. All of the results obtained by the proposed model and FEM (both the piezoelectric and thermoelastic cases), illustrated in Figure 2, Figure 3, Figure 4 and Figure 5, agree well with each other, verifying the effectiveness of the proposed model.

### 4.2. Effect of Sliding Speed

**Figure 6:**
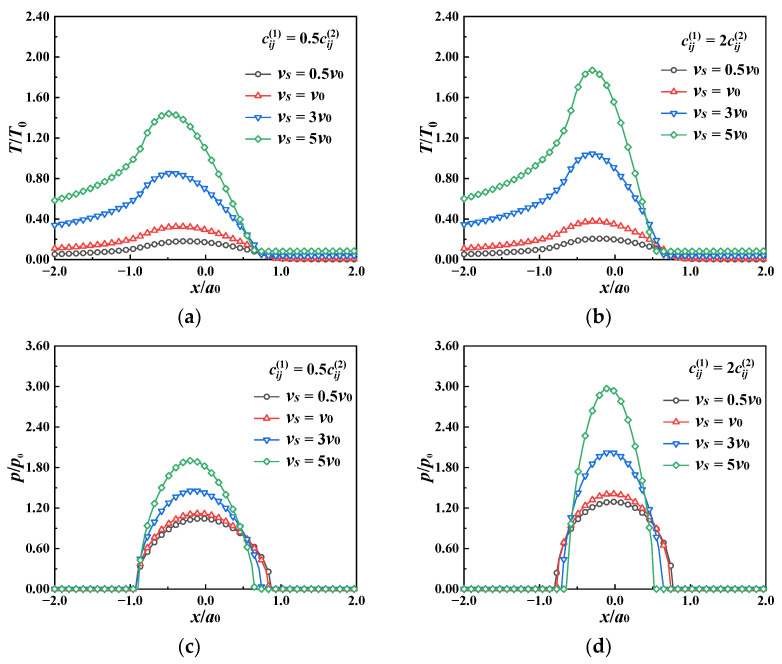
Temperature rise and contact pressure at the coating surface (the *x* axis) under different sliding speeds for “soft/hard” coating. (**a**) Temperature rise, $c_{ij}^{\left(1\right)}/c_{ij}^{\left(2\right)}=0.5$, (**b**) temperature rise, $c_{ij}^{\left(1\right)}/c_{ij}^{\left(2\right)}=2$, (**c**) contact pressure $c_{ij}^{\left(1\right)}/c_{ij}^{\left(2\right)}=0.5$, (**d**) contact pressure, $c_{ij}^{\left(1\right)}/c_{ij}^{\left(2\right)}=2$.

**Figure 7:**
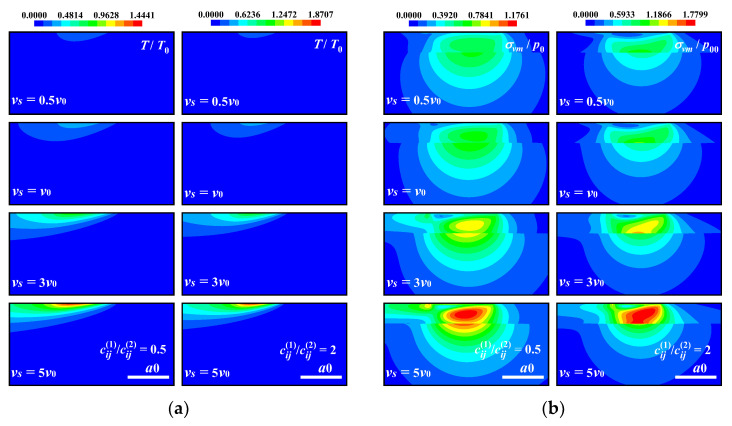
Temperature rise and von Mises stress on the *x*–*z* plane under different sliding speeds for “soft/hard” coating. (**a**) Temperature rise, (**b**) von Mises stress.

**Figure 8:**
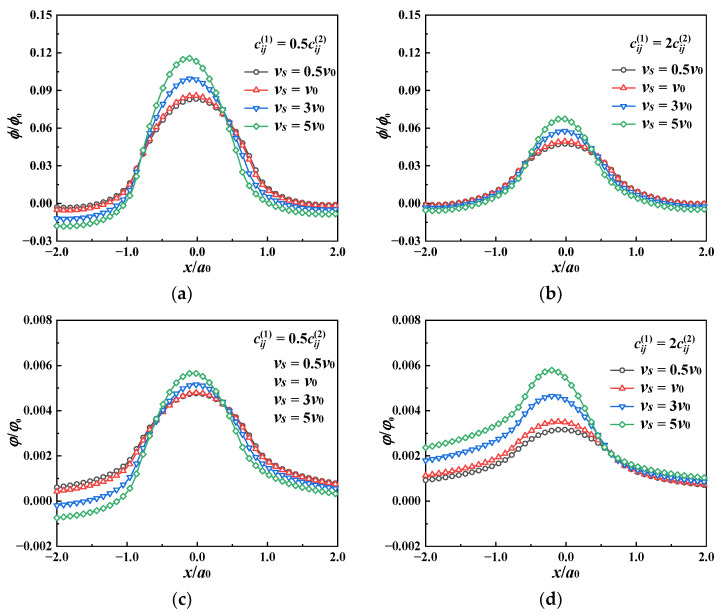
Electric and magnetic potentials at the coating surface (the *x* axis) under different sliding speeds for “soft/hard” coating. (**a**) Electric potential, $c_{ij}^{\left(1\right)}/c_{ij}^{\left(2\right)}=0.5$, (**b**) electric potential, $c_{ij}^{\left(1\right)}/c_{ij}^{\left(2\right)}=2$, (**c**) magnetic potential $c_{ij}^{\left(1\right)}/c_{ij}^{\left(2\right)}=0.5$, (**d**) magnetic potential $c_{ij}^{\left(1\right)}/c_{ij}^{\left(2\right)}=2$.

**Figure 9:**
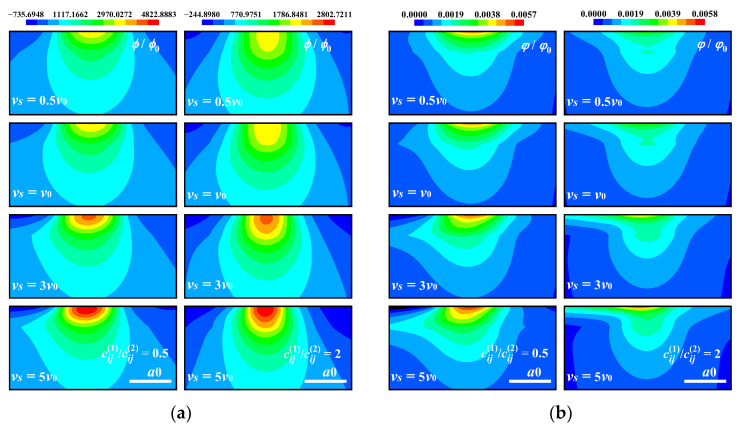
Electric and magnetic potentials of half-space on the *x*–*z* plane under different sliding speeds for “soft/hard” coating. (**a**) Electric potential, (**b**) magnetic potential.

The relative sliding velocity of the loaded ball is one of the key factors determining frictional heat flux. Its effects on the thermal contact behavior of the MEE material are explored, including the temperature rise, stress, and electric and magnetic potential distributions. Two types of coatings with different material parameters, $c_{ij}^{\left(1\right)}/c_{ij}^{\left(2\right)}=0.5$ and $c_{ij}^{\left(1\right)}/c_{ij}^{\left(2\right)}=2$, are designed, while the remaining material parameters are set to be the same as those of the substrate. The relative sliding velocity of the loaded ball is allowed to vary from 0.1 *v*_0_ to 5 *v*_0_. The frictional coefficient *μ_f_* = 0.2 and the simulated results are illustrated in Figure 6, Figure 7, Figure 8 and Figure 9.

It can be seen from Figure 6 that as the relative sliding velocity of the loaded ball increases, the frictional heat flux grows, which leads to an augmentation in the surface temperature rise and contact pressure for both the soft ($c_{ij}^{\left(1\right)}/c_{ij}^{\left(2\right)}=0.5$) and hard coating ($c_{ij}^{\left(1\right)}/c_{ij}^{\left(2\right)}=2$) cases. The temperature rise of the hard coating surface is slightly larger than that of the soft coating. This may be due to the higher contact pressure in the hard coating, accompanied by greater heat flux.

Figure 7 shows the temperature rise and contact pressure distribution for soft/hard coatings subjected to different sliding speeds. The faster the sliding speed, the more the surface temperature rises. The difference in the temperature rise distribution in different coatings is not obvious in the present cases, while some remarkable differences can be noticed in the von Mises stress contours for dissimilar coating materials. Stress discontinuity at the interface between the coating and the substrate exists for all cases. Stress concentration occurs in the coating in the soft coating case, but across the interface in the hard coating case.

The effects of the sliding speed on the electric and magnetic fields are shown in Figure 8 and Figure 9. In both the soft and hard coating cases, as the sliding speed increases, the contact pressure becomes higher, resulting in a rise in the surface electric and magnetic potentials within the contact area. The soft coating has higher electric and magnetic potentials than the hard coating, which means that the former has better piezomagnetic and piezoelectric performance. Regarding those outside of the contact area (*x* ≥ |*a*_0_|), in the soft coating case, an augmentation in the temperature rise makes the surface electric and magnetic potentials decrease slightly. However, the magnetic potential gradually increases at *x* ≥ *a*_0_ (see Figure 8). As shown in Figure 9, the electric and magnetic potentials in the coating material increase gradually with the sliding speed. Moreover, in the soft coating case, the electric potential is more concentrated on the contact surface, while, in the hard coating case, the magnetic potential is more concentrated on the contact surface.

### 4.3. Effect of Heat Conductivity and Thermal Modulus of Coating

**Figure 10:**
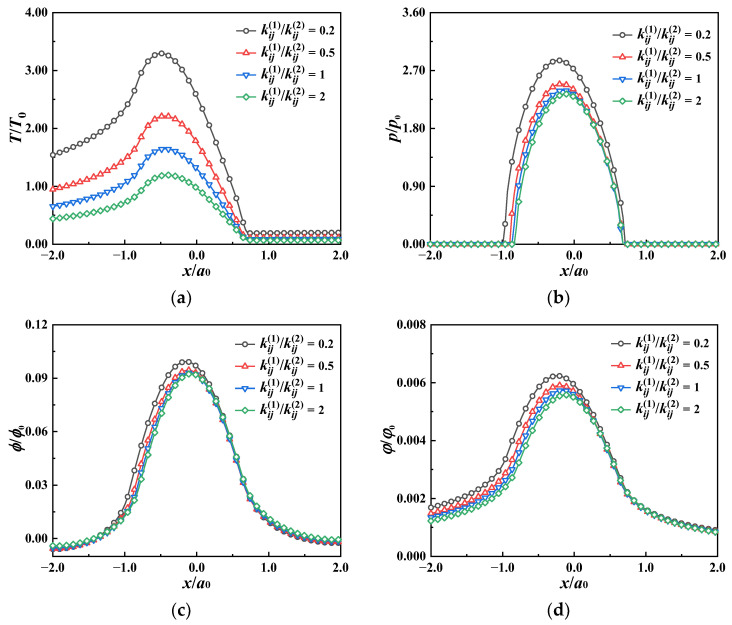
Contact behavior at the coating surface (the *x* axis) for MEE coatings of different thermal conductivities. (**a**) Temperature rise, (**b**) contact pressure, (**c**) electric potential, (**d**) magnetic potential.

The effect of the heat conductivity of the coating on the contact performance is investigated by changing the thermal conductivity ratio of the coating to the substrate, $k_{ij}^{\left(1\right)}/k_{ij}^{\left(2\right)}$, from 0.2 to 2, while the other material properties of the coating are identical to those of the substrate. The loaded ball slides on the coating surface with a velocity of *v*_s_ = 5 *v*_0_ = 5 m/s. Figure 10 demonstrates the temperature, pressure, and electric and magnetic potential distributions on the coating surface with different coating thermal conductivities. The surface temperature decreases when the coating thermal conductivity becomes larger, as well as the contact pressure. Similar to the sliding speed, the surface thermal expansion resulting from the larger temperature rise leads to a slight increase in the contact pressure when the coating thermal conductivity is small (see Figure 10b). The temperature rise decreases with the coating thermal conductivity, which causes the surface electric potential (−*a*_0_ < *x* < 0) and the magnetic potential (*x* < 0) to decrease slightly, while they scarcely change in other regions, as exhibited in Figure 10c,d.

**Figure 11:**
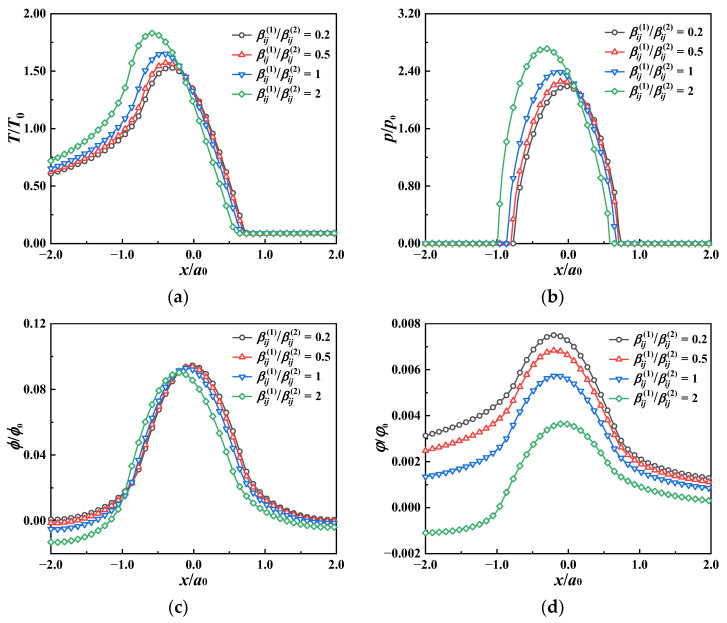
Contact behavior at the coating surface (the *x* axis) for MEE coatings of different thermal moduli. (**a**) Temperature rise, (**b**) contact pressure, (**c**) electric potential, (**d**) magnetic potential.

The ratio of the thermal modulus of the coating to the substrate $\beta_{ij}^{\left(1\right)}/\beta_{ij}^{\left(2\right)}$ ranges from 0.2 to 2. The effects of the coating thermal modulus on the temperature, pressure, and electric and magnetic potential are portrayed in Figure 11. As the coating thermal modulus increases, the surface temperature and the contact pressure are augmented remarkably for the latter part of the contact area along the sliding direction, while the variation trend is the opposite and slight in the former part of the contact area. Although the surface electric potential shares similar regularity with the temperature and the contact pressure, the effect of the coating thermal modulus is more obvious in the former part of the contact area. It is noted that the increase in the coating thermal modulus leads to a reduction in the magnetic potential across the whole surface.

### 4.4. Effect of Film Thickness

**Figure 12:**
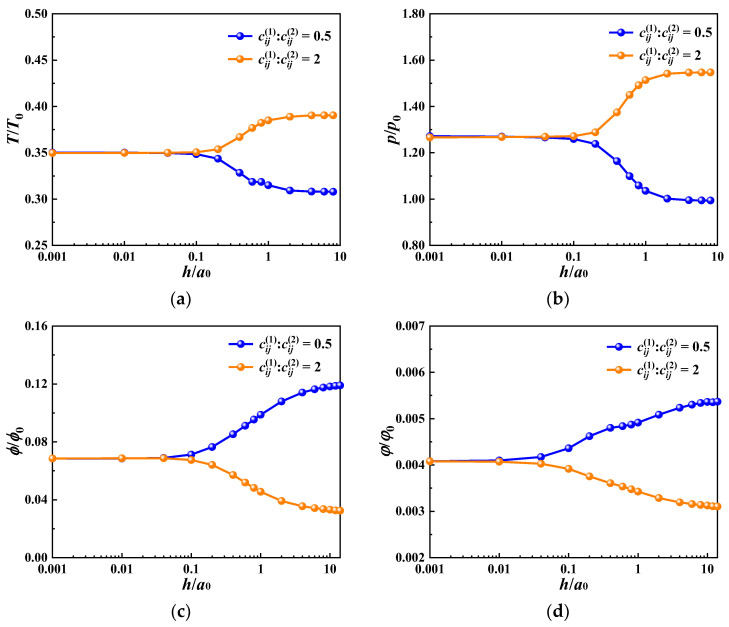
Effect of coating thickness on the contact behavior of MEE coating. (**a**) The maximum temperature rise; (**b**) the maximum contact pressure; (**c**) the maximum electric potential; (**d**) the maximum magnetic potential.

In order to study the effect of the coating thickness on the thermal contact behavior of the MEE material, the thicknesses of the soft and hard coatings are set to be 0.001 *a*_0_ to 14 *a*_0_, and the sliding speed *v*_s_ = *v*_0_ = 1 m/s. The rest of the coating parameters are set to be the same as those of the substrate. The calculation results of the maximum temperature rise, contact pressure, and electric potential and magnetic potential in the coating surface are shown in Figure 12. When the coating thickness is between ~0.1 *a*_0_ and 1 *a*_0_, the maximum surface temperature rise and the contact pressure are greatly affected by the thickness changes. Both the temperature rise and contact pressure in the hard coating case ($c_{ij}^{\left(1\right)}/c_{ij}^{\left(2\right)}=2$) are higher than those in the soft coating case ($c_{ij}^{\left(1\right)}/c_{ij}^{\left(2\right)}=0.5$). Correspondingly, when adjusting the thickness between ~0.003 *a*_0_ and 10 *a*_0_, the amplitudes of the electric potential and the magnetic potential change prominently. The electric potential and magnetic potential of the soft coating are higher than those of the hard coating. This phenomenon indicates that the temperature rise, contact pressure, electric potential, and magnetic potential on the surface of the MEE material can be controlled by adjusting the thickness of the coating material within a certain range (0.1 *a*_0_–1*a*_0_ for the temperature rise and the contact pressure; 0.003 *a*_0_–14 *a*_0_ for the electric and magnetic potential).

## 5. Conclusions

In the present work, a thermal contact model between a sliding ball and a coated MEE medium is established. To this end, the Fourier transform is performed on the general solutions of the magneto-electro-thermo-elastic field and then a set of analytical FRFs for the coated medium are derived. CGM and the DC-FFT algorithm are employed to enhance the proposed model. Furthermore, the proposed model is verified by comparing the results with those from the FEM (thermal case and piezoelectric case). A series of parametric studies are carried out with the proposed model, leading to the following conclusions.

1. As the sliding velocity increases, there is almost no difference in the temperature rise between the soft and hard coatings. The contact pressure increases more acutely for the material with a hard coating. For the electric and magnetic fields, both the electric and magnetic potentials increase gradually in the contact area. Outside of the contact area, the electric potential and the magnetic potential in the soft coating surface decrease slightly, but, in the hard coating surface, the electric potential decreases and the magnetic potential increases. In addition, the temperature and electric and magnetic potentials are continuous, and they are more concentrated in the soft coating. Meanwhile, the von Mises stress is discontinuous and is higher in the hard coating.
2. The greater the ratio of the thermal conductivity of the coating to that of the substrate, the lower the surface temperature rise, contact pressure, and electric and magnetic potentials. However, when the ratio increases, the surface temperature rise and the contact pressure increase, the magnetic potential decreases, and the electric potential only shifts slightly, with its value almost unchanged.
3. When the coating thickness increases within a certain range, the surface’s maximum temperature rises, and the contact pressure of the soft coating gradually decreases and is lower than that of the hard coating. The maximum electric and magnetic potentials in the soft coating case are augmented and are higher than those in the hard coating case. Moreover, when the coating thickness is smaller or greater than a certain range, the change in coating thickness has almost no effect on the MEE system.

## Data Availability

Data are contained within the article.

## Appendix A

The intermediate variables defined in Equation (16) have the following forms:

(A1) $$\begin{array}{l} \varpi_{1k}=a_{1}\alpha^{6}s_{k}^{6}-b_{1}\alpha^{6}s_{j}^{4}+f_{1}\alpha^{6}s_{j}^{2}-g_{1}\alpha^{6},\;\\ \varpi_{15}=a_{1}r^{6}s_{5}^{6}-b_{1}\alpha^{4}r^{4}s_{5}^{4}+f_{1}\alpha^{6}r^{2}s_{5}^{2}-g_{1}\alpha^{6},\;\\ \varpi_{2k}=a_{2}\alpha^{6}s_{k}^{6}-b_{2}\alpha^{6}s_{k}^{4}+f_{2}\alpha^{6}s_{k}^{2}-g_{2}\alpha^{6},\;\\ \varpi_{25}=a_{2}r^{6}s_{5}^{6}-b_{2}\alpha^{4}r^{4}s_{5}^{4}+f_{2}\alpha^{6}r^{2}s_{5}^{2}-g_{2}\alpha^{6},\;\\ \varpi_{3k}=a_{3}\alpha^{6}s_{k}^{6}-b_{3}\alpha^{6}s_{k}^{4}+f_{3}\alpha^{6}s_{k}^{2}-g_{3}\alpha^{6},\;\\ \varpi_{35}=a_{3}r^{6}s_{5}^{6}-b_{3}\alpha^{4}r^{4}s_{5}^{4}+f_{3}\alpha^{6}r^{2}s_{5}^{2}-g_{3}\alpha^{6},\;\\ \varpi_{4k}=a_{4}\alpha^{6}s_{k}^{6}-b_{4}\alpha^{6}s_{k}^{4}+f_{4}\alpha^{6}s_{k}^{2}-g_{4}\alpha^{6},\;\\ \varpi_{45}=a_{4}r^{6}s_{5}^{6}-b_{4}\alpha^{4}r^{4}s_{5}^{4}+f_{4}\alpha^{6}r^{2}s_{5}^{2}-g_{4}\alpha^{6},\;\\ \varpi_{55}=n_{0}r^{8}s_{5}^{8}-n_{1}\alpha^{2}r^{6}s_{5}^{6}+n_{2}\alpha^{4}r^{4}s_{5}^{4}-n_{3}\alpha^{6}r^{2}s_{5}^{2}+n_{4}\alpha^{8}, \end{array}$$

The constants in Equations (17)–(20) are expressed as follows:

(A2) $$\begin{array}{l} \kappa_{1k}=c_{13}\alpha^{2}s_{k}^{2}\varpi_{2k}+e_{31}\alpha^{2}s_{k}^{2}\varpi_{3k}+q_{31}\alpha^{2}s_{k}^{2}\varpi_{4k},\;\\ \kappa_{15}=c_{13}r^{2}s_{5}^{2}\varpi_{25}+e_{31}r^{2}s_{5}^{2}\varpi_{35}+q_{31}r^{2}s_{5}^{2}\varpi_{45},\\ \kappa_{2k}=c_{13}\alpha^{2}\varpi_{1k}+c_{33}\alpha^{2}s_{k}^{2}\varpi_{2k}+e_{33}\alpha^{2}s_{k}^{2}\varpi_{3k}+q_{33}\alpha^{2}s_{k}^{2}\varpi_{4k},\;\\ \kappa_{25}=c_{13}\alpha^{2}\varpi_{15}+c_{33}r^{2}s_{5}^{2}\varpi_{25}+e_{33}r^{2}s_{5}^{2}\varpi_{35}+q_{33}r^{2}s_{5}^{2}\varpi_{45},\\ \kappa_{3k}=c_{44}\alpha s_{k}\varpi_{1k}-c_{44}\alpha s_{k}\varpi_{2k}-e_{15}\alpha s_{k}\varpi_{3k}-q_{15}\alpha s_{k}\varpi_{4k},\;\\ \kappa_{35}=c_{44}rs_{5}\varpi_{15}-c_{44}rs_{5}\varpi_{25}-e_{15}rs_{5}\varpi_{35}-q_{15}rs_{5}\varpi_{45},\;\\ \kappa_{4k}=e_{15}\alpha s_{k}\varpi_{1k}-e_{15}\alpha s_{k}\varpi_{2k}+\varepsilon_{11}\alpha s_{k}\varpi_{3k}+d_{11}\alpha s_{k}\varpi_{4k},\;\\ \kappa_{45}=e_{15}rs_{5}\varpi_{15}-e_{15}rs_{5}\varpi_{25}+\varepsilon_{11}rs_{5}\varpi_{35}+d_{11}rs_{5}\varpi_{45},\;\\ \kappa_{5k}=e_{31}\alpha^{2}\varpi_{2k}+e_{33}\alpha^{2}s_{k}^{2}\varpi_{2k}-\varepsilon_{33}\alpha^{2}s_{k}^{2}\varpi_{3k}-d_{33}\alpha^{2}s_{k}^{2}\varpi_{4k},\;\\ \kappa_{55}=e_{31}\alpha^{2}\varpi_{25}+e_{33}r^{2}s_{5}^{2}\varpi_{25}-\varepsilon_{33}r^{2}s_{5}^{2}\varpi_{35}-d_{33}r^{2}s_{5}^{2}\varpi_{45},\\ \kappa_{6k}=q_{15}\alpha s_{k}\varpi_{1k}-q_{15}\alpha s_{k}\varpi_{2k}+d_{11}\alpha s_{k}\varpi_{3k}+\mu_{11}\alpha s_{k}\varpi_{4k},\;\\ \kappa_{65}=q_{15}rs_{5}\varpi_{15}-q_{15}rs_{5}\varpi_{25}+d_{11}rs_{5}\varpi_{35}+\mu_{11}rs_{5}\varpi_{45},\;\\ \kappa_{7k}=q_{31}\alpha^{2}\varpi_{2k}+q_{33}\alpha^{2}s_{k}^{2}\varpi_{2k}-d_{33}\alpha^{2}s_{k}^{2}\varpi_{3k}-\mu_{33}\alpha^{2}s_{k}^{2}\varpi_{4k},\;\\ \kappa_{75}=q_{31}\alpha^{2}\varpi_{2j}+q_{33}r^{2}s_{5}^{2}\varpi_{25}-d_{33}r^{2}s_{5}^{2}\varpi_{35}-\mu_{33}r^{2}s_{5}^{2}\varpi_{45}, \end{array}$$

The constant submatrices in Equation (30) are given as follows:

(A3) $$U=\left[\begin{array}{cccc} 1 & 0 & 0 & 0\\ 0 & 1 & 0 & 0\\ 0 & 0 & 1 & 0\\ 0 & 0 & 0 & -1 \end{array}\right],N_{1k}^{\left(j\right)}=\left[\begin{array}{cccc} \kappa_{21}^{\left(j\right)} & \kappa_{22}^{\left(j\right)} & \kappa_{23}^{\left(j\right)} & \kappa_{24}^{\left(j\right)}\\ \kappa_{51}^{\left(j\right)} & \kappa_{52}^{\left(j\right)} & \kappa_{53}^{\left(j\right)} & \kappa_{54}^{\left(j\right)}\\ \kappa_{71}^{\left(j\right)} & \kappa_{72}^{\left(j\right)} & \kappa_{73}^{\left(j\right)} & \kappa_{74}^{\left(j\right)}\\ \kappa_{31}^{\left(j\right)} & \kappa_{32}^{\left(j\right)} & \kappa_{33}^{\left(j\right)} & \kappa_{34}^{\left(j\right)} \end{array}\right]$$

(A4) $$N_{2k}^{\left(j\right)}=\left[\begin{array}{cccc} s_{1}^{\left(j\right)}\varpi_{21}^{\left(j\right)} & s_{2}^{\left(j\right)}\varpi_{22}^{\left(j\right)} & s_{3}^{\left(j\right)}\varpi_{23}^{\left(j\right)} & s_{4}^{\left(j\right)}\varpi_{24}^{\left(j\right)}\\ s_{1}^{\left(j\right)}\varpi_{31}^{\left(j\right)} & s_{2}^{\left(j\right)}\varpi_{32}^{\left(j\right)} & s_{3}^{\left(j\right)}\varpi_{33}^{\left(j\right)} & s_{4}^{\left(j\right)}\varpi_{34}^{\left(j\right)}\\ s_{1}^{\left(j\right)}\varpi_{41}^{\left(j\right)} & s_{2}^{\left(j\right)}\varpi_{42}^{\left(j\right)} & s_{3}^{\left(j\right)}\varpi_{43}^{\left(j\right)} & s_{4}^{\left(j\right)}\varpi_{44}^{\left(j\right)}\\ \varpi_{11}^{\left(j\right)} & \varpi_{12}^{\left(j\right)} & \varpi_{13}^{\left(j\right)} & \varpi_{14}^{\left(j\right)} \end{array}\right]$$

(A5) $$W_{0}^{}=\left[\begin{array}{c} -\tilde{\tilde{p}}-\left(\kappa_{25}^{\left(1\right)}-\beta_{3}^{\left(1\right)}\varpi_{55}^{\left(1\right)}\right)\left(A_{5}^{\left(1\right)}+\bar{A}{}_{5}^{\left(1\right)}\right)\\ -{\tilde{\tilde{q}}}_{b}-\left(\kappa_{55}^{\left(1\right)}+p_{3}^{\left(1\right)}\varpi_{55}^{\left(1\right)}\right)\left(A_{5}^{\left(1\right)}+\bar{A}{}_{5}^{\left(1\right)}\right)\\ -{\tilde{\tilde{g}}}_{b}-\left(\kappa_{75}^{\left(1\right)}+\lambda_{3}^{\left(1\right)}\varpi_{55}^{\left(1\right)}\right)\left(A_{5}^{\left(1\right)}+\bar{A}{}_{5}^{\left(1\right)}\right)\\ -in\mu_{f}\tilde{\tilde{p}}/\alpha^{2}-\kappa_{35}^{\left(1\right)}\left(A_{5}^{\left(1\right)}-\bar{A}{}_{5}^{\left(1\right)}\right) \end{array}\right],$$

(A6) $$W_{1}^{}=\left[\begin{array}{c} \left(\kappa_{25}^{\left(2\right)}-\beta_{3}^{\left(2\right)}\varpi_{55}^{\left(2\right)}\right)A_{5}^{\left(2\right)}-\left(\kappa_{25}^{\left(1\right)}-\beta_{3}^{\left(1\right)}\varpi_{55}^{\left(1\right)}\right)\left(A_{5}^{\left(1\right)}e^{-r_{1}s_{5}^{\left(1\right)}h_{1}}+\bar{A}{}_{5}^{\left(1\right)}e^{r_{1}s_{5}^{\left(1\right)}h_{1}}\right)\\ \left(\kappa_{55}^{\left(2\right)}+p_{3}^{\left(2\right)}\varpi_{55}^{\left(2\right)}\right)A_{5}^{\left(2\right)}-\left(\kappa_{55}^{\left(1\right)}+p_{3}^{\left(1\right)}\varpi_{55}^{\left(1\right)}\right)\left(A_{5}^{\left(1\right)}e^{-r_{1}s_{5}^{\left(1\right)}h_{1}}+\bar{A}{}_{5}^{\left(1\right)}e^{r_{1}s_{5}^{\left(1\right)}h_{1}}\right)\\ \left(\kappa_{75}^{\left(2\right)}+\lambda_{3}^{\left(2\right)}\varpi_{55}^{\left(2\right)}\right)A_{5}^{\left(2\right)}-\left(\kappa_{75}^{\left(1\right)}+\lambda_{3}^{\left(1\right)}\varpi_{55}^{\left(1\right)}\right)\left(A_{5}^{\left(1\right)}e^{-r_{1}s_{5}^{\left(1\right)}h_{1}}+\bar{A}{}_{5}^{\left(1\right)}e^{r_{1}s_{5}^{\left(1\right)}h_{1}}\right)\\ \kappa_{35}^{\left(2\right)}A_{5}^{\left(2\right)}-\kappa_{35}^{\left(1\right)}\left(A_{5}^{\left(1\right)}e^{-r_{1}s_{5}^{\left(1\right)}h_{1}}-\bar{A}{}_{5}^{\left(1\right)}e^{r_{1}s_{5}^{\left(1\right)}h_{1}}\right) \end{array}\right],$$

(A7) $$W_{2}=\left[\begin{array}{c} r_{2}s_{5}^{\left(2\right)}\varpi_{25}^{\left(2\right)}A_{5}^{\left(2\right)}/\alpha -r_{1}s_{5}^{\left(1\right)}\varpi_{25}^{\left(1\right)}\left(A_{5}^{\left(1\right)}e^{-r_{1}s_{5}^{\left(1\right)}h_{1}}-\bar{A}{}_{5}^{\left(1\right)}e^{r_{1}s_{5}^{\left(1\right)}h_{1}}\right)/\alpha \\ r_{2}s_{5}^{\left(2\right)}\varpi_{35}^{\left(2\right)}A_{5}^{\left(2\right)}/\alpha -r_{1}s_{5}^{\left(1\right)}\varpi_{35}^{\left(1\right)}\left(A_{5}^{\left(1\right)}e^{-r_{1}s_{5}^{\left(1\right)}h_{1}}-\bar{A}{}_{5}^{\left(1\right)}e^{r_{1}s_{5}^{\left(1\right)}h_{1}}\right)/\alpha \\ r_{2}s_{5}^{\left(2\right)}\varpi_{45}^{\left(2\right)}A_{5}^{\left(2\right)}/\alpha -r_{1}s_{5}^{\left(1\right)}\varpi_{45}^{\left(1\right)}\left(A_{5}^{\left(1\right)}e^{-r_{1}s_{5}^{\left(1\right)}h_{1}}-\bar{A}{}_{5}^{\left(1\right)}e^{r_{1}s_{5}^{\left(1\right)}h_{1}}\right)/\alpha \\ \varpi_{15}^{\left(2\right)}A_{5}^{\left(2\right)}/\alpha^{2}-\varpi_{15}^{\left(1\right)}\left(A_{5}^{\left(1\right)}e^{-r_{1}s_{5}^{\left(1\right)}h_{1}}+\bar{A}{}_{5}^{\left(1\right)}e^{r_{1}s_{5}^{\left(1\right)}h_{1}}\right)/\alpha^{2} \end{array}\right]$$

## Appendix B

The process of determining the unknowns of the temperature solution ($A_{5}^{\left(1\right)}$, $\bar{A}{}_{5}^{\left(1\right)}$, $A_{5}^{\left(2\right)}$) is to solve the linear equations (Equation (24)) as follows:

(A8) $$\left[\begin{array}{ccc} 1 & -1 & 0\\ \frac{\varpi_{55}^{\left(1\right)}\theta_{5}}{\varpi_{55}^{\left(2\right)}} & \frac{\varpi_{55}^{\left(1\right)}}{\varpi_{55}^{\left(2\right)}\theta_{5}} & -1\\ \chi_{2}\theta_{5} & -\chi_{2}/\theta_{5} & -1 \end{array}\right]\left[\begin{array}{c} A_{5}^{\left(1\right)}\\ \bar{A}{}_{5}^{\left(1\right)}\\ A_{5}^{\left(2\right)} \end{array}\right]=\left[\begin{array}{c} \frac{\tilde{\tilde{q}}}{k_{33}^{\left(1\right)}r_{1}s_{5}^{\left(1\right)}\varpi_{55}^{\left(1\right)}}\\ 0\\ 0 \end{array}\right].$$

Equation (A8) can be written as

(A9) $$A_{5}^{\left(1\right)}-\bar{A}{}_{5}^{\left(1\right)}=\frac{\tilde{\tilde{q}}}{k_{33}^{\left(1\right)}r_{1}s_{5}^{\left(1\right)}\varpi_{55}^{\left(1\right)}},$$

(A10) $$\frac{\varpi_{55}^{\left(1\right)}\theta_{5}}{\varpi_{55}^{\left(2\right)}}A_{5}^{\left(1\right)}+\frac{\varpi_{55}^{\left(1\right)}}{\varpi_{55}^{\left(2\right)}\theta_{5}}\bar{A}{}_{5}^{\left(1\right)}-A_{5}^{\left(2\right)}=0,$$

(A11) $$\chi_{2}\theta_{5}A_{5}^{\left(1\right)}-\chi_{2}/\theta_{5}\bar{A}{}_{5}^{\left(1\right)}-A_{5}^{\left(2\right)}=0.$$

Equation (A10) is multiplied by $\chi_{2}$ minus Equation (A11) multiplied by $\frac{\varpi_{55}^{\left(1\right)}}{\varpi_{55}^{\left(2\right)}}$, resulting in

(A12) $$\left(\frac{\varpi_{55}^{\left(1\right)}}{\varpi_{55}^{\left(2\right)}}-\chi_{2}\right)\theta_{5}A_{5}^{\left(1\right)}+\left(\frac{\varpi_{55}^{\left(1\right)}}{\varpi_{55}^{\left(2\right)}}+\chi_{2}\right)/\theta_{5}\bar{A}{}_{5}^{\left(1\right)}=0.$$

Simultaneously, Equation (A9) and Equation (A12) are grouped:

(A13) $$A_{5}^{\left(1\right)}-\bar{A}{}_{5}^{\left(1\right)}=\frac{\tilde{\tilde{q}}}{k_{33}^{\left(1\right)}r_{1}s_{5}^{\left(1\right)}\varpi_{55}^{\left(1\right)}},$$

(A14) $$\left(\frac{\varpi_{55}^{\left(1\right)}}{\varpi_{55}^{\left(2\right)}}-\chi_{2}\right)\theta_{5}A_{5}^{\left(1\right)}+\left(\frac{\varpi_{55}^{\left(1\right)}}{\varpi_{55}^{\left(2\right)}}+\chi_{2}\right)/\theta_{5}\bar{A}{}_{5}^{\left(1\right)}=0.$$

Equation (A10) is multiplied by $\left(\frac{\varpi_{55}^{\left(1\right)}}{\varpi_{55}^{\left(2\right)}}+\chi_{2}\right)/\theta_{5}$ plus Equation (A12), leading to

(A15) $$A_{5}^{\left(1\right)}=\frac{\left(\varpi_{55}^{\left(1\right)}+\varpi_{55}^{\left(2\right)}\chi_{2}\right)\tilde{\tilde{q}}}{k_{33}^{\left(1\right)}r_{1}s_{5}^{\left(1\right)}\varpi_{55}^{\left(1\right)}\left[\left(\varpi_{55}^{\left(1\right)}+\chi_{2}\varpi_{55}^{\left(2\right)}\right)+\left(\varpi_{55}^{\left(1\right)}-\chi_{2}\varpi_{55}^{\left(2\right)}\right)\theta_{5}^{2}\right]}.$$

After obtaining $A_{5}^{\left(1\right)}$, $\bar{A}{}_{5}^{\left(1\right)}$ can be obtained by Equation (A9):

(A16) $$\bar{A}{}_{5}^{\left(1\right)}=-\frac{\left(\varpi_{55}^{\left(1\right)}-\varpi_{55}^{\left(2\right)}\chi_{2}\right)\theta_{5}^{2}}{\varpi_{55}^{\left(1\right)}+\varpi_{55}^{\left(2\right)}\chi_{2}}A_{5}^{\left(1\right)}.$$

After obtaining $A_{5}^{\left(1\right)}$ and $\bar{A}{}_{5}^{\left(1\right)}$, $A_{5}^{\left(2\right)}$ is easy to obtain from Equation (A10) or Equation (A11):

(A17) $$A_{5}^{\left(2\right)}=\chi_{2}\theta_{5}A_{5}^{\left(1\right)}-\frac{\chi_{2}}{\theta_{5}}A_{5}^{\left(1\right)}$$
